# Changes in physical activity and adiposity with all-cause, cardiovascular disease, and cancer mortality

**DOI:** 10.1038/s41366-022-01195-z

**Published:** 2022-08-01

**Authors:** Matthew N. Ahmadi, I-Min Lee, Mark Hamer, Borja del Pozo Cruz, Li Jung Chen, Elif Eroglu, Yun-Ju Lai, Po Wen Ku, Emmanuel Stamatakis

**Affiliations:** 1 Charles Perkins Centre, Faculty of Medicine and Health, School of Health Sciences, The University of Sydney, Sydney, NSW Australia; 2 Brigham and Women’s Hospital and Harvard Medical School, Harvard T.H. Chan School of Public Health, Boston, MA USA; 3 Division of Surgery and Interventional Science, Faculty Medical Sciences University College London, London, UK; 4 Center for Active and Healthy Ageing, Southern Denmark University, Odense, Denmark; 5 Department of Exercise Health Science, National Taiwan University of Sport, Taichung, Taiwan; 6 Division of Endocrinology and Metabolism, Department of Internal Medicine, Puli Branch of Taichung Veterans General Hospital, Taichung, Taiwan; 7 School of Medicine, National Yang-Ming University, Taipei, Taiwan; 8 Graduate Institute of Sports and Health Management, National Chung Hsing University, Taichung, Taiwan; 9 Department of Kinesiology, National Tsing Hua University, Hsinchu, Taiwan

**Keywords:** Risk factors, Diseases

## Abstract

**Background:**

The relationship between joint changes in physical activity and adiposity with mortality is not well understood. We examined the association of changes in these two established risk factors with all-cause (ACM), cardiovascular disease (CVD), and cancer mortality.

**Methods:**

We used longitudinal data from Taiwan’s MJ Cohort, comprising 116,228 general population adults recruited from 1998-2013 with repeated measures 4.6 y (2.5) apart and followed up for mortality for 11.9 y (3.5). Physical activity, body mass index (BMI), waist circumference (WC), and body fat percentage (BF%) groups and changes were based on public health and clinical guidelines.

**Results:**

Compared to stable-insufficient physical activity, increasing physical activity from any baseline level was associated with lower ACM (HR [95%CI]): 0.85 [0.74, 0.96]) and CVD mortality (0.72 [0.55, 0.93]) risk. This was approximately equal to meeting physical activity guidelines at both timepoints (eg: 0.71 [0.58, 0.88] for CVD mortality). Compared to stable-overweight/moderate adiposity, decreasing adiposity level attenuated but did not offset mortality risk for all three outcomes (eg: BMI = 0.95 [0.76, 1.16] for CVD mortality). Only maintaining a healthy adiposity level at both timepoints offset mortality risk (BMI = 0.75 [0.61, 0.89]) for CVD mortality). In the joint changes analyses, lower mortality risk was a consequence of increases in physical activity across adiposity change groups (eg: WC decrease = 0.57 [0.48, 0.67]; WC stability = 0.73 [0.66, 0.80], WC increase = 0.83 [0.72, 0.97] for ACM). Decreasing adiposity attenuated the negative associations of decreased physical activity (BF% = 1.13 [0.95, 1.35] for ACM).

**Conclusions:**

We found a lower risk for ACM, CVD, and cancer mortality from increasing physical activity and an attenuation from decreasing adiposity regardless of baseline levels. The beneficial associations of joint changes were primarily driven by physical activity, suggesting lower mortality risk may be more immediate through physical activity improvements compared to adiposity improvements alone.

## Introduction

The negative health consequences of excess adiposity, including increased risk of premature mortality and noncommunicable disease such as cardiovascular disease (CVD) and cancer [1], are widely recognized. Similarly, physical inactivity increases risk of a long list of adverse health outcomes, including CVD and premature mortality [2]. Studies that examined the joint associations between baseline physical activity and adiposity with mortality suggest that physical activity may attenuate the risk of premature death but does not always eliminate the increased risk associated with excess adiposity [3–8].

Evidence investigating the joint associations of physical activity and adiposity on mortality has almost exclusively relied on single baseline assessments. This is problematic because both physical activity and adiposity will change over time [9, 10]. Thus, evidence based on single baseline measurements is susceptible to nondifferential misclassification and regression dilution bias due to within-person variability over the follow up period. This can make it difficult to discern nuanced attributes of each risk factor [11, 12]. A recent analysis of the UK Biobank reported positive changes in physical activity or adiposity attenuated the deleterious associations of negative changes in the other risk factor [13]. Such findings contrast with baseline assessment-only studies, that are limited by the absence of re-examination assessments, reporting either an absence of a synergistic relationship or that associations of physical activity and mortality were independent of obesity status [7, 14–16].

Based on systematic reviews and meta-analyses, the 2020 WHO Physical Activity and Sedentary Behaviour Guidelines [2] and the Physical Activity Guidelines for Americans, 2nd Edition [17] reported there was insufficient evidence to determine the longitudinal relationship of physical activity and adiposity with mortality and whether this varied by ethnicity. Further, it is not known whether there are differential associations for changes in physical activity and adiposity over time with cause specific mortality related to CVD or cancer.

Longitudinal studies on physical activity and adiposity are of particular importance among Asian countries where there is a greater predisposition to cardiometabolic disorders than western countries for a given adiposity level [18, 19] and where cancer [20] has become the leading cause of death. Both the European Society of Cardiology Clinical Practice Guidelines Committee [21] and American College of Cardiology/American Heart Association Task Force on Clinical Practice Guidelines [22] identified a need to investigate long-term changes in CVD risk factors. Such information is needed improve personalized treatments among high-risk groups, including those with Asian ethnicity. In a population cohort of Taiwanese adults, we examined the associations of physical activity and adiposity changes with ACM, CVD, and cancer mortality.

## Methods

### Study design

We used data from the Taiwan MJ cohort, comprising adults undergoing routine health screening at the MJ Health Management Institution [23, 24]. Participants have provided informed consent and our study was approved by the National Changhua University of Education Research Ethics Committee, Taiwan, China (NCUEREC-108-072). During each assessment, participants underwent physical examinations by trained practitioners, provided blood samples after overnight fasting for 12–14 hours, and completed questionnaires on lifestyle and health behaviours (physical activity, dietary habits, smoking, alcohol consumption, hour spent sleeping). Hypertension and diabetes diagnosis was diagnosed through medical history or during the physical examination (hypertension = blood pressure ≥140/90 mmHg [25]) or fasting blood sample (diabetes ≥ 126 mg/dL). In this study, we included participants who were 18 y or older between 1998–2013, had at least one re-examination assessment ≥2 y after their baseline assessment, and complete data. To minimize the potential influence of reverse causality, we excluded participants who were underweight at baseline assessment (18.5 kg/m^2^), had CVD or cancer diagnosis at baseline or at re-examination assessment, and those who died within the first two years of follow-up (Supplementary Fig. 1).

### Physical activity assessment

Participants completed a questionnaire where they reported their physical activity duration and types (eg: gardening, basketball, swimming, etc.). Activities were classified as: light (2.5 METs), moderate (4.5 METs), medium-vigorous (6.5 METs), or high-vigorous (8.5 METs) intensity based on the Physical Activity Compendium [26]. To account for variations of the questionnaire that were used for follow up waves, we used baseline and follow up assessments that included the same questionnaire version. The questions, data handling, and construct and face validity are shown in Supplementary Text 1. We calculated physical activity volume (MET-h) per week by multiplying activity intensity (MET) by duration (h). We categorised physical activity around the current WHO guidelines as: inactive (<1 MET-h): insufficient (> 1 to <7.5 MET-h); and sufficient (≥7.5 MET-h). Physical activity changes were categorised as decreased (moved category downward), stable (stayed in the same category) or increased (moved category upward).

### Adiposity indicator assessment

Body weight (kg), height (cm), body fat (BF%), and waist circumference (cm) were measured according to standardized procedures without shoes by trained staff [27]. Weight and height were measured with a Nakamura KN-5000A auto-anthropometer (Nakamura, Tokyo, Japan) to the nearest 0.1 kg and 0.1 cm. Waist circumference was measured at the midway point between the inferior margin of the last rib and the crest of the ilium in a horizontal plane to the nearest 0.1 cm. Based on the Western Pacific Region of WHO recommendations [28, 29], body mass index (BMI) was classified as obese (≥ 25 kg/m^2^), overweight (≥ 23 to <25 kg/m^2^), or healthy (≥ 18.5 to <23 kg/m^2^). BF% was measured using a bioelectrical impedance analysis instrument (InBody Co., Ltd., Seoul, Korea). In the absence of widely accepted population-based risk categories or cut-offs for BF%, groups were based on sex-specific BF% distributions using BMI categories in accordance with prior published research [30] and classified as: high (≥25.2% for men and ≥34.8% for women), moderate (<25.2% to ≥21.6% for men and <34.8% to ≥31.0% for women), and low (<21.6% for men and <31.0% for women). Based on WHO [31] and IDF [32] recommendations, waist circumference (WC) was classified as: high (≥85 cm for men and ≥80 cm for women), moderate (<85 cm to <75 cm for men and <80 cm to >70 cm for women) and low (≥75 cm for men and ≥70 cm for women). BMI, BF%, and WC changes were categorised as decreased (moved category downward), stable (stayed in the same category) or increased (moved category upward).

#### Joint physical activity and adiposity exposure

Participants were classified into 1 of 9 mutually-exclusive groups based on their change in physical activity (decreased, stable, increased) and adiposity indicator (decreased, stable, increased) between baseline and re-examination assessment.

### Mortality ascertainment

Participants included in all analyses were followed up for mortality through the National Death file [23] until death or censoring (31st October 2019). Time to event or censoring began counting after the re-examination assessment. CVD mortality included deaths from coronary heart disease (ICD-9 = 410–414 and 420–429; ICD-10 = I20–I25), stroke (ICD-9 = 430–438; ICD-10 = I60–I69) and other circulatory diseases (ICD-9 = 390–392, 393–398, 401–405 and 440; ICD-10 = I10–I15, I01–I02.0, I05–I09, I27, I30–I52, I70, and I71). For cancer mortality, we used codes 140–208 of ICD-9 and codes C00-C97 of ICD-10.

### Statistical analysis

Hazard ratios (HR) and 95% confidence interval ((95% CI) for ACM were estimated for each of the joint exposure groups, using Cox proportional hazards regression models. We used Fine-Gray subdistribution hazard models to estimate HR and 95% CI for CVD and cancer, where mortality from other causes were considered a competing risk [33]. For independent analyses of physical activity and adiposity indicators, the referent group was insufficient-stable physical activity or overweight/moderate-stable adiposity. For joint analyses, the referent group was stable physical activity and stable adiposity. Effect modification was tested by fitting an interaction term between physical activity and adiposity groups. Follow-up years was used as the timescale and we adjusted all models for baseline and re-examination covariates of: age, sex, smoking status, alcohol consumption, diet, education, and sleep. Baseline physical activity and adiposity were included as covariates in the joint analyses. Complete covariate definitions are provided in Supplementary Table 1. For all sets of analyses, we calculated E-values to estimate the plausibility of bias from unmeasured confounding. The E-values indicate the minimum strength of association that an unmeasured confounder would need with both exposure and outcome to explain away the observed association [34, 35]. We also used a negative control outcome of accidents/sequelae of transport or other accidents that do not have a mechanistic link to physical activity and adiposity. Negative controls can improve causal inference by illustrating pervasive bias and confounding [36]. If the negative control has a similar association pattern as the primary outcomes, then it is more plausible associations are due to bias and confounding than causal mechanisms. We performed additional analyses by imputing missing data for covariates by using multiple imputation by chained equations (5 imputed datasets) for the association of joint changes and ACM. We also included analyses adjusting for potential mediators that included hypertension and diabetes diagnosis. Since physical activity and adiposity maybe mutual effect modifiers of each other, we repeated the separate association analyses with mutual adjustment. Due to the relatively young age of the cohort, we performed sensitivity analyses of only participants who were ≥40 years at baseline assessment for all joint analyses.

We performed all analyses using R statistical software with the rms and survival packages [37, 38]. We reported this study as per the Strengthening the Reporting of Observational Studies in Epidemiology (STROBE) guideline (Supplementary STROBE Statement).

## Results

Among 116,228 participants (46.2% female), 3838 deaths occurred (607 and 1777 due to CVD and cancer). There was an average of 4.6 (2.5)y between baseline and re-examination assessments and 11.9 (3.5)y to mortality/censoring corresponding to 1,384,723 person-years. Participant characteristics by physical activity and BMI change categories is presented in Table 1. Supplementary Table 2 presents participant lifestyle and health characteristics at the re-examination assessment. Among physical activity change groups, 17,513 (15.1%), 60,388 (51.9%), and 38,327 (33.0%) decreased, maintained, and increased their levels, respectively. Among BMI change groups, 8004 (6.9%), 87,507 (75.3%), and 20,717 (17.8%) decreased, maintained, and increased their levels. Supplementary Table 3 shows the interquartile range of physical activity and adiposity changes between assessments. Supplementary Fig 2 displays the degree of change or stability by baseline categories for physical activity, BMI, WC, and BF%. Characteristics of excluded participants are shown in Supplementary Tables 4 and 5.

**Table 1:** Participant descriptive characteristics by physical activity and body mass index change groups (*n* = 116,228).

Physical activity		Decrease			Stable			Increase		
BMI		Decrease	Stable	Increase	Decrease	Stable	Increase	Decrease	Stable	Increase
Total										
*n* (%)	116,228 (100)	1125 (1.0)	13,022 (11.2)	3366 (2.9)	4115 (3.5)	45,920 (39.5)	10,353 (8.9)	2764 (2.4)	28,565 (24.6)	6998 (6.0)
Female, *n* (%)	53,671 (46.2)	480 (42.7)	5748 (44.1)	1242 (36.9)	1589 (38.6)	21,154 (46.1)	4348 (42.0)	1186 (42.9)	14,581 (51.0)	3343 (47.8)
Age	38.0 (11.7)	40.9 (13.9)	38.1 (12.6)	34.6 (12.1)	41.3 (12.8)	38.6 (11.8)	35.9 (11.6)	40.0 (11.3)	37.7 (10.7)	35.6 (10.2)
Follow-up, years	11.9 (3.5)	11.7 (3.8)	12.0 (3.9)	12.0 (3.5)	11.7 (3.5)	11.7 (3.6)	11.7 (3.3)	12.0 (3.3)	12.1 (3.1)	12.0 (2.7)
Education, *n* (%)										
No schooling	2471 (2.1)	44 (3.9)	333 (2.6)	44 (1.3)	107 (2.6)	947 (2.1)	154 (1.5)	77 (2.8)	653 (2.3)	112 (1.6)
Elementary	9209 (7.9)	160 (14.2)	1182 (9.1)	264 (7.8)	441 (10.7)	3509 (7.6)	660 (6.4)	280 (10.1)	2231 (7.8)	482 (6.9)
Junior High	5645 (4.9)	78 (6.9)	779 (6.0)	173 (5.1)	214 (5.2)	2237 (4.9)	436 (4.2)	134 (4.8)	1297 (4.5)	297 (4.2)
Senior High	24,523 (21.1)	245 (21.8)	2787 (21.4)	781 (23.2)	872 (21.2)	9138 (19.9)	2164 (20.9)	605 (21.9)	6263 (21.9)	1668 (23.8)
Vocational	28,562 (24.6)	187 (16.6)	3027 (23.2)	797 (23.7)	840 (20.4)	10,957 (23.9)	2634 (25.4)	642 (23.2)	7466 (26.1)	2012 (28.8)
College/ University	32,733 (28.2)	282 (25.1)	3488 (26.8)	949 (28.2)	1085 (26.4)	13,558 (29.5)	3117 (30.1)	687 (24.9)	7755 (27.1)	1812 (25.9)
Graduate School	13,085 (11.3)	129 (11.5)	1426 (11.0)	358 (10.6)	556 (13.5)	5574 (12.1)	1188 (11.5)	339 (12.3)	2900 (10.2)	615 (8.8)
Smoking status, *n* (%)										
Never	79,156 (68.1)	779 (69.2)	8757 (67.2)	2234 (66.4)	2782 (67.6)	31,500 (68.6)	6915 (66.8)	1838 (66.5)	19,655 (68.8)	4696 (67.1)
2nd hand	6395 (5.5)	51 (4.5)	688 (5.3)	194 (5.8)	204 (5.0)	2496 (5.4)	615 (5.9)	130 (4.7)	1592 (5.6)	425 (6.1)
Former	6958 (6.0)	78 (6.9)	815 (6.3)	212 (6.3)	317 (7.7)	2774 (6.0)	574 (5.5)	199 (7.2)	1624 (5.7)	365 (5.2)
Frequently	4385 (3.8)	44 (3.9)	563 (4.3)	132 (3.9)	161 (3.9)	1766 (3.8)	412 (4.0)	108 (3.9)	974 (3.4)	225 (3.2)
Daily	19,334 (16.6)	173 (15.4)	2199 (16.9)	594 (17.6)	651 (15.8)	7384 (16.1)	1837 (17.7)	489 (17.7)	4720 (16.5)	1287 (18.4)
Alcohol consumption, *n* (%)										
None	96,245 (82.8)	904 (80.4)	10,643 (81.7)	2772 (82.4)	3330 (80.9)	37,777 (82.3)	8475 (81.9)	2298 (83.1)	24,160 (84.6)	5886 (84.1)
Former	2483 (2.1)	27 (2.4)	335 (2.6)	72 (2.1)	107 (2.6)	930 (2.0)	225 (2.2)	53 (1.9)	577 (2.0)	157 (2.2)
1-2 times/wk	11,872 (10.2)	131 (11.6)	1423 (10.9)	365 (10.8)	449 (10.9)	4872 (10.6)	1139 (11.0)	270 (9.8)	2578 (9.0)	645 (9.2)
3-4 times/wk	3882 (3.3)	41 (3.6)	431 (3.3)	106 (3.1)	158 (3.8)	1631 (3.6)	355 (3.4)	91 (3.3)	850 (3.0)	219 (3.1)
Daily	1746 (1.5)	22 (2.0)	190 (1.5)	51 (1.5)	71 (1.7)	710 (1.5)	159 (1.5)	52 (1.9)	400 (1.4)	91 (1.3)
Diet^a^	2.1 (1.23)	2.1 (1.3)	2.1 (1.2)	2.1 (1.2)	2.1 (1.2)	2.0 (1.2)	2.0 (1.2)	2.0 (1.2)	1.9 (1.1)	1.9 (1.1)
Sleep, *n* (%)^b^										
≤4 h	895 (0.8)	10 (0.9)	111 (0.9)	25 (0.7)	40 (1.0)	368 (0.8)	73 (0.7)	21 (0.8)	196 (0.7)	51 (0.7)
>4 to ≤ 6 h	21,982 (18.9)	210 (18.7)	2494 (19.2)	666 (19.8)	806 (19.6)	8822 (19.2)	2016 (19.5)	570 (20.6)	5130 (18.0)	1268 (18.1)
>6 to ≤ 8 h	84,724 (72.9)	828 (73.6)	9440 (72.5)	2417 (71.8)	2954 (71.8)	33,551 (73.1)	7486 (72.3)	1967 (71.2)	20,952 (73.3)	5129 (73.3)
>8 h	8627 (7.4)	77 (6.8)	977 (7.5)	258 (7.7)	315 (7.7)	3179 (6.9)	778 (7.5)	206 (7.5)	2287 (8.0)	550 (7.9)
Hypertension, *n* (%)	5478 (4.7)	76 (6.8)	711 (5.5)	100 (3.0)	272 (6.6)	2383 (5.2)	363 (3.5)	150 (5.4)	1235 (4.3)	188 (2.7)
Diabetes, *n* (%)	1699 (1.5)	36 (3.2)	231 (1.8)	32 (1.0)	119 (2.9)	712 (1.6)	93 (0.9)	76 (2.7)	358 (1.3)	42 (0.6)
Baseline, Median [IQR]										
Physical Activity^c^	3.2 [0.0, 6.8]	4.7 [3.7, 11.7]	4.7 [3.7, 11.7]	5.7 [4.8, 12.7]	3.7 [1.2, 13.7]	3.7 [1.2, 8.7]	3.7 [1.2, 8.7]	1.2 [0.0, 2.2]	1.8 [0.0, 2.7]	0.6 [0.0, 1.2]
Body mass Index^d^	22.8 [20.8, 25.0]	24.6 [23.4, 25.4]	22.6 [20.6, 25.7]	22.6 [21.6, 23.9]	24.5 [23.4, 25.4]	22.5 [20.5, 25.6]	22.6 [21.7, 23.9]	24.6 [23.4, 25.5]	22.2 [20.4, 25.5]	22.6 [21.6, 23.9]
Body fat percent	25.5 [22.0, 29.7]	27.9 [23.6, 32.9]	25.4 [21.7, 29.5]	23.6 [20.3, 28.2]	27.3 [23.5, 32.4]	25.6 [22.1, 29.5]	24.3 [21.1, 28.8]	28.2 [24.2, 33.3]	25.7 [22.4, 29.8]	25.0 [21.7, 29.5]
Waist Circumference^e^	76.0 [70.0, 84.0]	81.0 [76.0, 85.0]	76.0 [70.0, 85.0]	75.0 [71.0, 80.0]	81.0 [76.0, 85.0]	76.0 [69.0, 85.0]	75.0 [70.0, 81.0]	81.0 [76.0, 86.0]	75.0 [69.0, 84.0]	75.0 [70.0, 80.0]
Follow-up, Median [IQR]										
Physical Activity^c^	3.7 [1.2, 8.7]	1.2 [0.6, 3.7]	1.2 [0.6, 3.7]	1.2 [0.6, 3.7]	3.7 [1.2, 13.7]	3.7 [1.2, 8.7]	2.7 [1.2, 8.7]	8.7 [2.1, 15.7]	3.7 [1.2, 9.7]	3.7 [1.2, 8.7]
Body mass Index^d^	23.2 [21.2, 25.5]	22.8 [22.2, 24.2]	22.7 [21.0, 26.1]	24.5 [23.4, 25.6]	22.8 [22.3, 24.2]	22.6 [20.9, 25.9]	24.3 [23.3, 25.5]	22.8 [22.2, 24.1]	22.5 [20.8, 25.7]	24.4 [23.3, 25.6]
Body fat percent	26.4 [22.7, 30.7]	25.1 [21.5, 29.5]	26.2 [22.5, 30.5]	26.9 [23.1, 32.2]	24.5 [21.0, 29.2]	26.2 [22.6, 30.2]	27.1 [23.0, 32.7]	25.1 [21.1, 29.3]	26.4 [22.9, 30.4]	28.2 [23.7, 33.6]
Waist Circumference^e^	78.0 [71.0, 85.0]	78.0 [73.0, 82.0]	78.0 [71.0, 86.0]	81.0 [76.0, 86.0]	78.0 [73.0, 82.0]	77.0 [70.0, 86.0]	81.0 [75.0, 86.0]	77.0 [72.0, 82.0]	76.0 [70.0, 85.0]	81.0 [75.0, 86.0]
Mortality rate/1000 person-years^f^										
All-cause	2.8	2.5	3.3	6.2	2.4	2.8	4.4	1.6	2.4	3.6
CVD	0.4	0.3	0.6	0.8	0.5	0.5	0.7	0.2	0.3	0.6
Cancer	1.3	1.1	1.4	2.6	1.1	1.3	1.9	0.8	1.2	1.6

^a^Fruits and vegetable servings/day.

^b^h/day.

^c^Units = Met-h/week.

^d^Units = Kg/m^2^.

^e^Units = centimetres.

^f^Calculated from Body Mass Index sample.

### Independent association of physical activity and adiposity changes with mortality

#### Physical activity

**Fig. 1:**
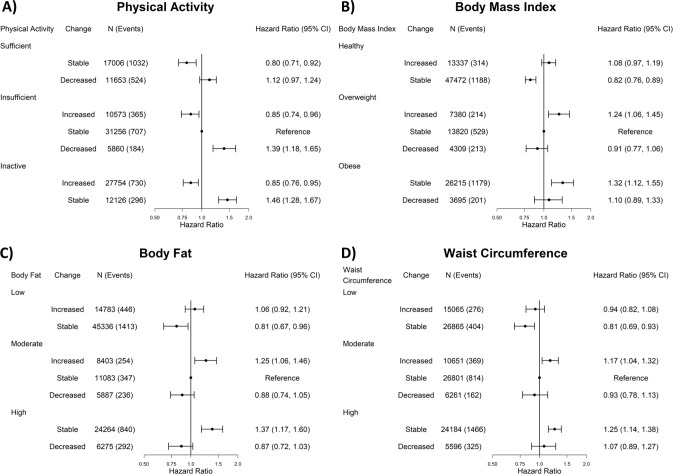
Independent association for physical activity, body fat percent, body mass index, and waist circumference changes with all-cause mortality (physical activity: *n* = 116,228, events = 3838; body fat percent: *n* = 116,031, events = 3828; body mass index: *n* = 116,228, events = 3838; waist circumference: *n* = 115,423, events = 3816). All results are adjusted for: age, sex, smoking status, alcohol consumption, sleep duration, diet (fruits and vegetables consumption), and education.

**Fig. 2:**
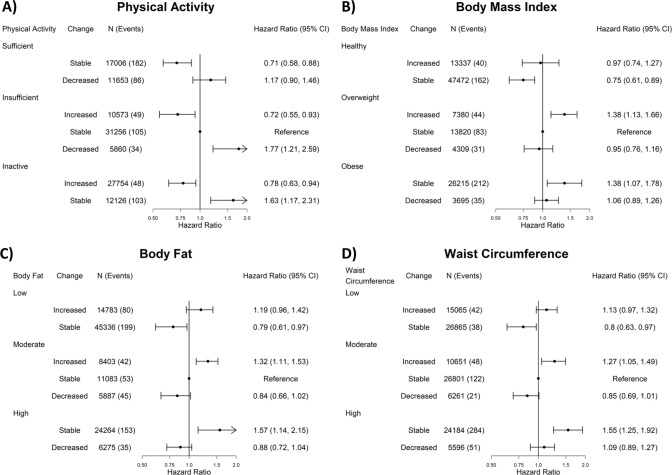
Independent association for physical activity, body fat, body mass index, and waist circumference changes with CVD mortality (physical activity: *n* = 116,228, events = 607; body fat percent: *n* = 116,031, events = 606; body mass index: *n* = 116,228, events = 607; waist circumference: *n* = 115,423, events = 606). All results are adjusted for: age, sex, smoking status, alcohol consumption, sleep duration, diet (fruits and vegetables consumption), and education.

The associations for independent changes from baseline to re-examination with ACM, CVD, and cancer are shown in Figs. 1, 2, and Supplementary Fig 3, respectively. Compared to stable-insufficient physical activity, increasing physical activity was consistently associated with lower mortality risk. For example, participants who were inactive or had insufficient physical activity at baseline and increased their physical activity had a 15% (HR [95%CI]: 0.85 [0.76, 0.95] for inactive-increased and 0.85 [0.74, 0.96] for insufficient-increased) lower risk of ACM (Fig. 1). For all three mortality outcomes, increasing physical activity was associated with a similar lower mortality risk as maintaining sufficient physical activity (eg: 0.80 [0.71, 0.92] for ACM). Decreasing physical activity from insufficient to become inactive was associated with a higher mortality risk that was similar in magnitude to being inactive at both timepoints for all three outcomes.

#### Adiposity

Increasing adiposity from a baseline level of overweight/moderate was detrimentally associated with all three mortality outcomes, compared to stable-overweight/moderate adiposity. We observed the highest association magnitude for CVD mortality. Relative to stable-overweight/moderate adiposity, for BMI, BF%, and WC, there was a 38% (1.38 [1.13, 1.66]), 32% (1.32 [1.11, 1.53]), and 27% (1.27 [1.05, 1.49]) higher CVD mortality risk, respectively (Fig. 2). Increasing adiposity from a baseline of overweight/moderate had a similar association magnitude as being/having obese/high adiposity at both timepoints for ACM and cancer mortality, whereas for CVD mortality maintaining obesity/high adiposity presented the highest risk (eg: 1.55 [1.25, 1.92] for waist circumference). Decreasing BMI from an overweight or obese level attenuated, but did not offset, mortality risk. Similar attenuations were observed for decreasing BF% and WC from moderate or high adiposity. For example, when waist circumference decreased, cancer mortality risk was 0.92 (0.68, 1.19) and 1.05 (0.87, 1.27) for moderate and high baseline levels (Supplementary Fig 3C).

### Joint association of physical activity and adiposity changes with mortality

#### All-cause mortality

**Fig. 3:**
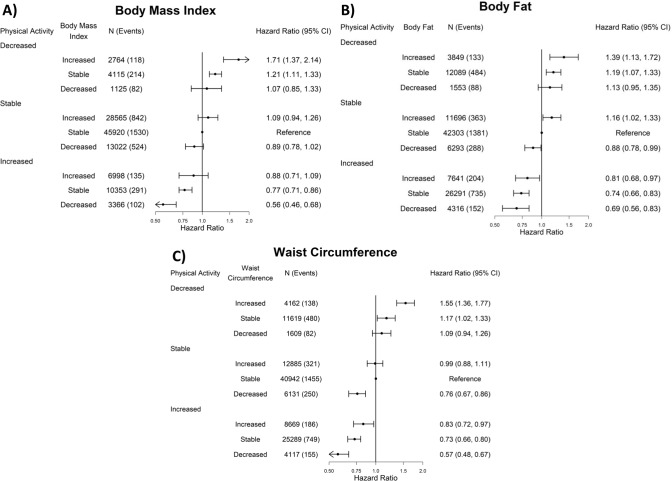
Joint association of physical activity and adiposity changes with all-cause mortality (body fat: *n* = 116,031, events = 3828; body mass index: *n* = 116,228, events = 3838; waist circumference: *n* = 115,423; events = 3816). All results are adjusted for: age, sex, baseline physical activity, baseline adiposity (body fat percentage, body mass index, or waist circumference), smoking status, alcohol consumption, sleep duration, diet (fruits and vegetables consumption), and education.

Increasing physical activity and decreasing adiposity was associated with substantially lower ACM risk compared to the stable physical activity-adiposity group (Fig. 3). The physical activity by adiposity interaction test was statistically significant for adiposity markers (*p* < 0.01). Increasing physical activity also offset the deleterious associations of increasing adiposity. For example, when physical activity increased, we did not observe higher ACM risk from increased BMI (0.88 [0.71, 1.09]). The risk was attenuated when the adiposity indicator was BF% (0.81 [0.68, 0.97]) and WC (0.83 [0.72, 0.97]). Similarly, decreasing adiposity attenuated the association between decreased physical activity and higher mortality risk. The attenuation was similar across BMI (1.07 [0.85, 1.33]), BF% (1.13 [0.95, 1.35]), and WC (1.09 [0.94, 1.26]). When physical activity was stable, ACM risk was not higher from increased adiposity, however when physical activity decreased there was a higher mortality risk when adiposity was stable (eg: BMI = 1.21 [1.11, 1.33]) or increased (eg: BMI = 1.71 [1.37, 2.14]).

#### Cardiovascular disease and cancer mortality

**Fig. 4:**
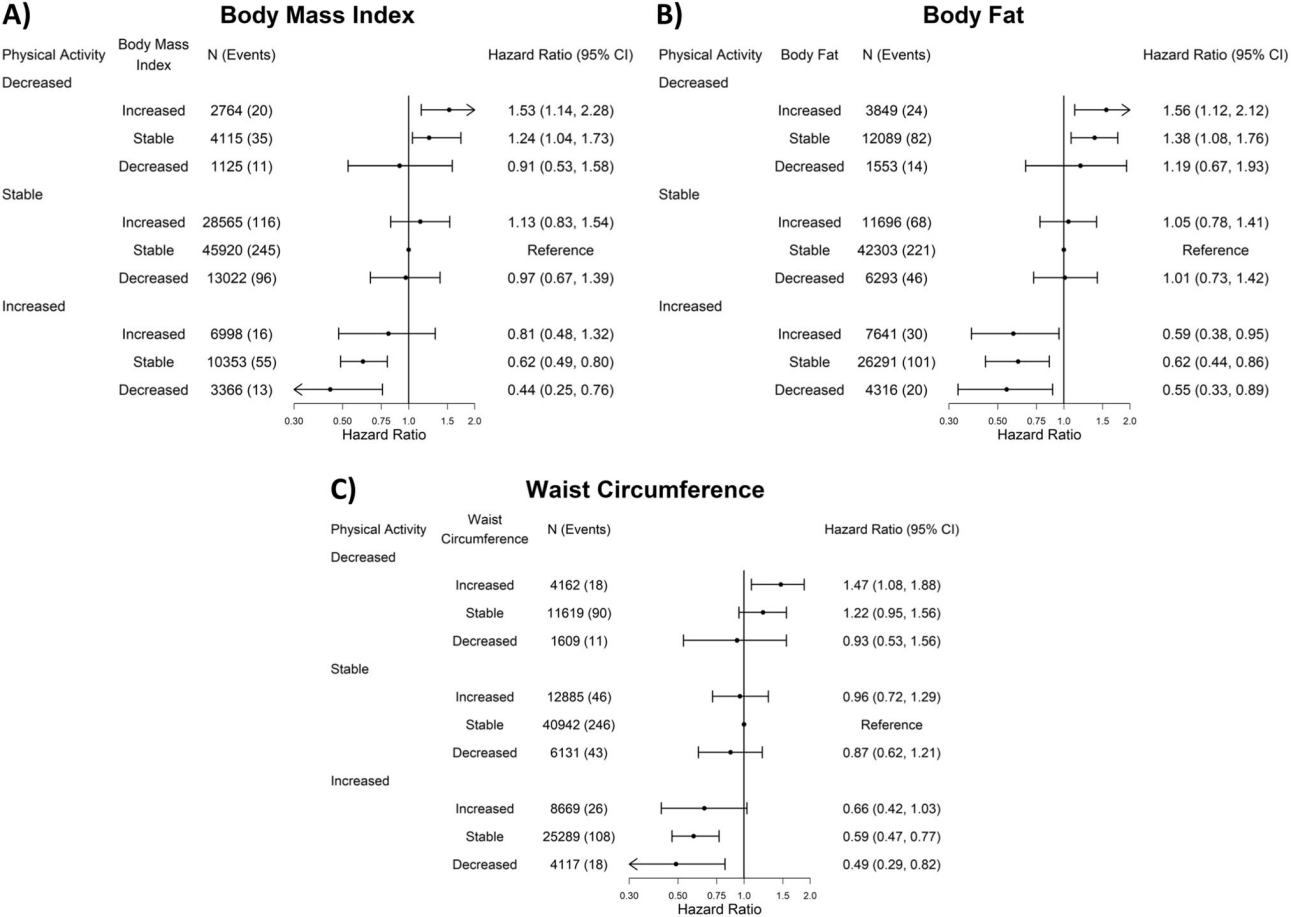
Joint association of physical activity and adiposity changes with cardiovascular disease mortality (body fat: *n* = 116,031, events = 606; body mass index: *n* = 116,228, events = 607; waist circumference: *n* = 115,423; events = 606). All results are adjusted for: age, sex, baseline physical activity, baseline adiposity (body fat percentage, body mass index, or waist circumference), smoking status, alcohol consumption, sleep duration, diet (fruits and vegetables consumption), and education.

For CVD mortality, we observed the most pronounced decreased hazard when physical activity increased and adiposity decreased for BMI (0.44 [0.25, 0.76]), BF% (0.55 [0.33, 0.89]), and WC (0.49 [0.29, 0.82]), relative to the stable physical activity-adiposity group (Fig. 4). Maintaining stable physical activity mitigated the associations of increases in all three adiposity markers (eg: BMI = 1.13 [0.83, 1.54]), whilst increasing physical activity reversed the association for BF% (0.59 [0.38, 0.95]; Fig. 4B). Conversely, maintaining stable BMI or BF% did not mitigate the associations of decreased physical activity. We did not find an association for higher CVD mortality from decreased physical activity when combined with decreased adiposity (eg: waist circumference = 0.93 [0.53, 1.56]), although with wide 95% CIs due to the low number of events in this group. We observed similar association patterns for cancer mortality (Supplementary Fig 4). Increased physical activity combined with decreased (eg: BMI = 0.58 [0.44, 0.77], Supplementary Fig 4A) or stable adiposity (BMI = 0.81 [0.69, 0.94]) was associated with the lowest cancer mortality risk across adiposity change groups. Decreasing and stable adiposity attenuated the association between decreased physical activity and cancer mortality, except for stable WC (1.19 [1.05, 1.35], Supplementary Fig 4C). The physical activity by adiposity interaction test was statistically significant for adiposity markers for CVD (*p* = 0.04) and cancer (*p* = 0.03) mortality.

#### Additional analyses

The E-values (Supplementary Tables 6–8) indicated a substantial degree of unmeasured confounding would be required to reduce the observed association to the null for increased physical activity and decreased adiposity. For example, the E-value for CVD mortality suggests that an unmeasured confounder would need to be associated with the joint physical activity-increase adiposity-decrease exposure and outcome with at least a 3.04 to 3.97-fold increase in risk to explain away the observed associations. The negative control association patterns suggest there were minimal confounding effects on the joint associations we observed (Supplementary Figs 5, 6). Restricting the joint analyses to participants who were ≥40 years at baseline or when multiple imputation was applied did not appreciably change the direction or magnitude of the association patterns (Supplementary Figs 7–10). Additional adjustment for hypertension and diabetes, and mutual adjustment for physical activity and adiposity did not materially change the associations (Supplementary Figs 11–16).

## Discussion

In our study examining changes in physical activity and adiposity with mortality, and in one of the largest longitudinal studies in an Asian population, we found positive changes in one risk factor (ie: physical activity or adiposity) attenuated and, in some cases, almost eliminated the deleterious association from negative changes in the other risk factor. The direction of the joint association was primarily driven by physical activity changes across BMI, BF%, and WC groups. We found that decreasing adiposity did not offset ACM risk in the presence of physical activity reductions. For most adiposity markers, increasing physical activity was associated with lower ACM and CVD mortality risk regardless of adiposity changes. Among physical activity change groups, increasing physical activity to meet guidelines lowered mortality risk by 15-28% and the magnitude was equal to meeting guidelines at both timepoints. Our results indicate positive health outcomes may be more pronounced and immediate through increases in physical activity whereas decreases in adiposity may have a longer induction period before health effects are observed.

Our results provide relevant information for clinicians and researchers who need to provide cost-effective interventions that require immediate maturation of health benefits, such as those counseling individuals who are at high-risk of a cardiovascular event. Such a finding calls for greater efforts by primary care services to support patients to increase their physical activity or to maintain compliance with guideline recommendations. This is especially pertinent in light of research showing physical inactivity has remained unchanged since 2000 in high income Asian countries [39] whilst heart disease [40] and cancer [41] incidence has increased. We found the positive associations for increasing physical activity were most pronounced for CVD mortality (22–28% lower mortality) and an increase in physical activity among the inactive baseline group lowered CVD mortality. The higher reduction in CVD mortality risk, compared to cancer and ACM risk is reflective of the direct effects physical activity has on cardiovascular health [42]. For example, an estimated 27% and 19% of the reduction in CVD rates can be explained by the beneficial effects of physical activity on hypertension and lipid profile [43]. Our findings substantiate the results of a prior study in the UK Biobank that also operationalized physical activity change according to guidelines [13] and other Western population studies that used trajectories or study specific change groupings [44–47].

It is possible the absence of significantly lower mortality risk from decreasing adiposity relative to stable-overweight/moderate adiposity is attributable to the higher risk for CVD [48] and certain cancers [49] among Asian populations compared to their counterparts from western populations for a given adiposity level. Our categories of adiposity change based on Asian-specific guidelines, may also indicate guidelines are imprecise with respect to the cut-off value for healthy adiposity status as it relates to mortality risk. This is consistent with a pooled analysis of 20 cohorts across seven countries in the Asia Cohort Consortium that reported the lowest CVD mortality risk for East Asians was between 20.0–22.4 kg/m^2^ which is lower than the current Asian specific cut-off of 23.0 kg/m^2^ recommended by the WHO [50]. Among 500,000 Chinese adults in the Kadoorie Biobank a similar absence of lower risk for multiple cancer sites has been reported for general and central adiposity levels that would be classified as healthy by current guidelines [51, 52]. In our study, such misclassification of adiposity-related health risks would have led to regression dilution contributing to an absence of lower CVD and cancer mortality risk. Collectively these findings suggest further refinement for appropriate adiposity cut-offs may be required to assess adiposity-related risks in East Asian populations.

We did not find evidence for the obesity paradox which has been reported in prior longitudinal studies among Asian [53–55] and Western [56, 57] populations. Our exclusion of participants who were underweight at baseline or had a history of major chronic disease prior to follow up assessment allowed us to decrease the possibility of reverse causation from prodromal illness that effect adiposity and physical activity levels. Our CVD and cancer results support the findings from a meta-analysis of randomized control trials in individuals with obesity that examined weight-loss and ACM but was underpowered to examine cause-specific mortality [58]. Population-level trials, such as The Look AHEAD Trial examining the effects of changes in risk factors with CVD mortality have low feasibility [59]. Event induction in the general population would require randomized controlled trials to be impracticably large to have sufficient power to assess CVD or cancer mortality.

The beneficial relationship we observed from joint changes in physical activity and adiposity are in contrast to baseline-only studies that reported an independence among the risk factors [6, 60, 61]. Baseline-only studies assume both risk factors stay constant over time, or if changes occur the rank order stays the same. Such assumptions are not pragmatic and contribute to association attenuation that make it difficult to discern the mutual contributions of physical activity and adiposity. In our study, positive changes in one risk factor mitigated the deleterious association of negative changes in the other risk factor, whilst positive changes in both was associated with the lowest mortality risk. Prior longitudinal studies using subjective cut-offs to define changes have reported increasing physical activity was not associated with lower mortality risk when combined with decreased adiposity [15, 62]. Our results indicate when guideline recommendations are used to define changes there is a beneficial relationship with mortality risk. We found physical activity to have a greater effect on the direction of the joint association than adiposity. This could be reflective of a longer latency period before adiposity changes become clinically evident relative to physical activity changes. Narrative reviews suggest increasing physical activity may lead to more dynamic metabolic adaptations, whereas the effects of adiposity changes on metabolic health accumulate gradually [63–65].

### Study strengths and limitations

We present a comprehensive analysis of joint physical activity and adiposity changes in a large population cohort with 12 years of follow-up. Our study is the first analysis of physical activity and adiposity with repeated measures and mortality outcomes, which directly addresses a major gap identified by the WHO’s recent guidelines of the need for longitudinal evidence from non-western countries [66]. We included general and central adiposity measures which provides important information for tailored interventions due to differences in health risks [67]. While we took extensive measures to reduce the possibility of reverse causation through undiagnosed/occult disease, we cannot rule out entirely reverse causation as a partial explanation of the our findings. Our operationalization of physical activity and adiposity changes based on guidelines allows for easier uptake and interpretation by clinicians and researchers in future longitudinal studies.

There were also several limitations that warrant consideration. First, our study was observational which precludes inferences about causality and residual confounding is possible. Our negative control associations and E-values, however, indicate that the effects of confounding on our findings were minimal. Second, we used two measures of each exposure and could not measure trajectory curves. Third, our physical activity estimates relied on self-reported data, however due to social desirability and recall bias the impact of nondifferential misclassification would have attenuated the associations. Self-reported physical activity accuracy can also be affected by age, sex, and socioeconomic status [68]. Thus, our results more likely underestimated the true importance of physical activity. The same is not true for our adiposity estimates that were objectively measured during assessment centre visits. The magnitude of associations reported for adiposity are more likely to reflect the true underlying association. Cardiorespiratory fitness, which has been shown to have associations with mortality risk among metabolically healthy individuals with obesity independent of physical activity levels [69–71], was not measured in the MJ Cohort. Some groupings in our negative control analysis had a low number of events, which may compromise the precision of the hazards attributed to the negative control outcome.

## Conclusions

Our findings provide novel evidence on the potential health consequences of independent and joint physical activity and adiposity changes in an under-studied Asian population. Our results suggest the association between physical activity and adiposity is driven by physical activity changes. Considering the absence of longitudinal studies assessing both physical activity and adiposity, our study provides support for public health physical activity guidelines. Promotion of physical activity is an important strategy, ancillary to decreasing adiposity, to attain immediate health improvement to prevent CVD, cancer, and premature mortality.

## Supplementary information

Supplemental Document

## Data Availability

The UK Biobank data that support the findings of this study can be accessed by researchers on application (https://www.ukbiobank.ac.uk/register-apply/).
