# Supplementary material for: Changes in physical activity and adiposity with all-cause, cardiovascular disease, and cancer mortality
Source: Int J Obes (Lond). 2022 Aug 1;46(10):1849–58. doi: 10.1038/s41366-022-01195-z (PMC9492547; doi:10.1038/s41366-022-01195-z)
Supplement: Supplementary file 1 — Supplemental Document [file 41366_2022_1195_MOESM1_ESM.docx]

**Changes in physical activity and adiposity with all-cause, cardiovascular disease, and cancer mortality**

**Supplemental Document**

| **Page** | **Item** |
| --- | --- |
| **1** | **Supplemental Figure 1**: Flow diagram of participants in the study |
| **2** | **Supplemental Table 1**: Covariate definitions |
| **4** | **Supplemental Table 2:** Participant lifestyle and health characteristics at follow up assessment |
| **6** | **Supplemental Table 3:** Physical activity and adiposity change values between baseline and follow-up assessments |
| **7** | **Supplemental Figure 2**: Degree of physical activity, body fat percent, body mass index, and waist circumference changes between baseline and follow up assessments |
| **8** | **Supplemental Table 4:** Characteristics of participants who were excluded from analyses |
| **10** | **Supplemental Table 5:** Baseline characteristics of participants who had a re-examination assessment and participants who only had a baseline assessment |
| **11** | **Supplemental Figure 3**: Independent association for physical activity, body fat, body mass index, and waist circumference changes with cancer mortality (physical activity: n=116,228, events=1,777; body fat percent: n=116,031, events=1,773; body mass index: n=116,228, events=1,777; waist circumference: n=115,423, events=1,767) |
| **12** | **Supplemental Figure 4**: Joint association of physical activity and adiposity changes with cancer mortality (body fat: n= 116,031, events= 1,773; body mass index: n=116,228, events= 1,777; waist circumference: n= 115,423; events= 1,767) |
| **13** | **Supplemental Table 6**: Independent physical activity change E-values for all-cause, cardiovascular disease, and cancer mortality |
| **14** | **Supplemental Table 7:** Independent adiposity change E-values for all-cause, cardiovascular disease, and cancer mortality |
| **15** | **Supplemental Table 8:** Joint physical activity and adiposity change E-values for all-cause, cardiovascular disease, and cancer mortality |
| **16** | **Supplemental Figure 5:** Independent association for physical activity, body fat percent, body mass index, and waist circumference with negative control outcome of accidents/sequelae of transport or other accidents (physical activity: n=116,228, events=132; body fat percent: n=116,031, events=132; body mass index: n=116,228, events=132; waist circumference: n=3,816, events=132) |
| **17** | **Supplemental Figure 6:** Joint association of physical activity and adiposity changes with negative control outcome of accidents/sequelae of transport or other accidents (body fat: n= 116,031, events= 132; body mass index: n=116,228, events= 132; waist circumference: n= 115,423; events= 132) |
| **18** | **Supplemental Figure 7:** Joint association of physical activity and adiposity changes with all-cause mortality among participants 40 years or older at baseline assessment (body mass index: n=43,107, events=3,165; body fat: n=43,038, events=3,160; waist circumference: n=42,881, events=3,150) |
| **19** | **Supplemental Figure 8:** Joint association of physical activity and adiposity changes with cardiovascular disease mortality among participants 40 years or older at baseline assessment (body mass index: n=43,107, events=529; body fat: n=43,038, events=528; waist circumference: n=42,881, events=528) |
| **20** | **Supplemental Figure 9:** Joint association of physical activity and adiposity changes with cancer mortality among participants 40 years or older at baseline assessment (body mass index: n=43,107, events=1,429; body fat: n=43,038, events=1,428; waist circumference: n=42,881, events=1,423 |
| **21** | **Supplemental Figure 10:** Joint association of physical activity and adiposity changes with all-cause mortality using non-imputed and imputed datasets |
| **22** | **Supplemental Figure 11:** Independent association for physical activity, body fat percent, body mass index, and waist circumference changes with all-cause mortality with adjustment for hypertension and diabetes, and mutual adjustment for physical activity and adiposity. |
| **23** | **Supplemental Figure 12:** Independent association for physical activity, body fat, body mass index, and waist circumference changes with CVD mortality with adjustment for hypertension and diabetes, and mutual adjustment for physical activity and adiposity. |
| **24** | **Supplemental Figure 13:** Independent association for physical activity, body fat, body mass index, and waist circumference changes with cancer mortality with adjustment for hypertension and diabetes, and mutual adjustment for physical activity and adiposity. |
| **25** | **Supplemental Figure 14:** Joint association of physical activity and adiposity changes with all-cause mortality with adjustment for hypertension and diabetes |
| **26** | **Supplemental Figure 15:** Joint association of physical activity and adiposity changes with cardiovascular disease mortality with adjustment for hypertension and diabetes |
| **27** | **Supplemental Figure 16:** Joint association of physical activity and adiposity changes with cancer mortality with adjustment for hypertension and diabetes |
| **28** | **Supplemental Text:** MJ Cohort physical activity questionnaire, construct validity, and face validity |
| **32** | **STROBE Statement** |

Supplemental Figure 1. Flow diagram of participants in the study


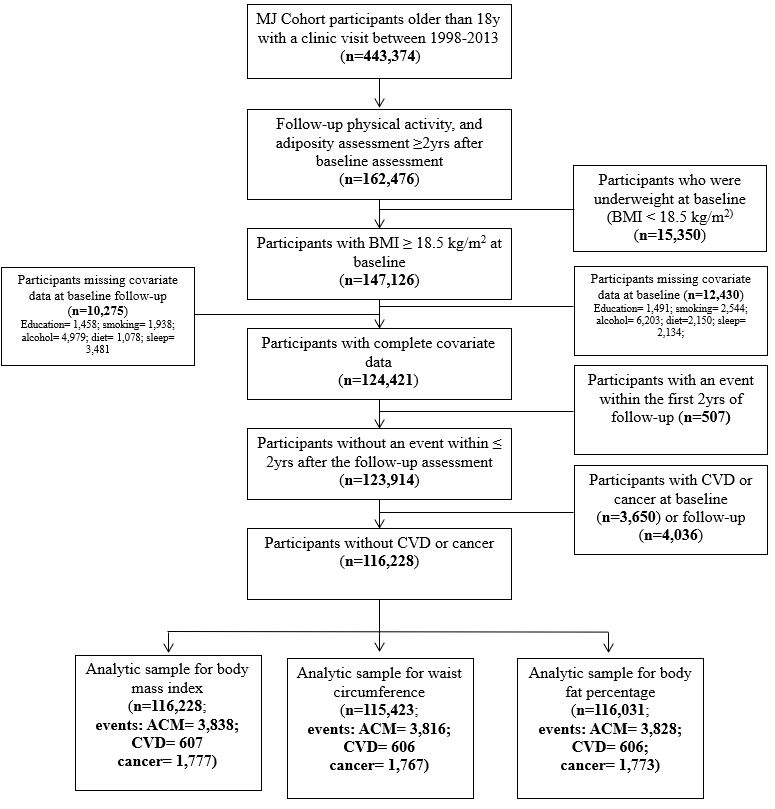


Supplemental Table 1: Covariate definitions

| **Variable** | **Definition** |
| --- | --- |
| Age | Continuous (years) |
| Sex | Female/Male |
| Smoking status | - Never, - 2^nd^ hand (someone in household smokes) - Quit, - Frequently (3-5 days/week), - Daily |
| Alcohol consumption | - Never - Former drinker - 1-2 days/week - 3-4 days/week - Daily |
| Sleep | - <=4 hours - >4 to <=6 hours - >6 to <= 8 hours - >8 hours |
| Diet | Fruits and vegetables servings/day |
| Education | - no schooling - elementary - junior-high - senior-high - vocational - college/university - graduate-school |
| Diabetes diagnosis | Yes/No, self-reported |
| Hypertension diagnosis | Yes/No, self-reported |
| Physical activity group (for independent association of adiposity baseline changes analyses) | - Inactive - Insufficient - Sufficient |
| Body mass index group (for independent association of adiposity baseline changes analyses) | - Healthy - Overweight - Obese |

Variables were treated as time-varying covariates

Diet, smoking status, and alcohol consumption were ascertained through the following questions:

| Question | Response |
| --- | --- |
| Diet |  |
| How many servings of green, leafy vegetables (like carrots, spinach, squash, tomatoes, and other dark green and yellow vegetables) do you eat? | Per day (continuous) |
| How many servings of fruits do you eat? (1 serving is equivalent to half a medium sized apple, grapefruit, or guava, 1 orange or kiwi, half a kilo of papaya, or watermelon, 5 lychees, 12 grapes, or dragon eye | Per day (continuous) |
| Smoking |  |
| Do you smoke? | Multiple choice:  -Do not smoke  -Has quit smoking  -Do not smoke but often inhale second-hand smoke  -1-3 times a week  -4-5 times a week  -Everyday |
| Alcohol consumption |  |
| Do you drink alcohol? | Multiple choice:  -No  -Quit drinking  -1-2 times a week  -3-4 times a week  -5-6 times a week  -Daily |

Supplemental Table 2: Participant lifestyle and health characteristics at follow up assessment

| **Physical Activity** |  | **Decrease** | | |  | **Stable** | | |  | **Increase** | | |
| --- | --- | --- | --- | --- | --- | --- | --- | --- | --- | --- | --- | --- |
| **BMI** |  | **Decrease** | **Stable** | **Increase** |  | **Decrease** | **Stable** | **Increase** |  | **Decrease** | **Stable** | **Increase** |
|  | **Total** |  |  |  |  |  |  |  |  |  |  |  |
| **n (%)** | 116,228 (100) | 1,125 (1.0) | 13,022 (11.2) | 3,366 (2.9) |  | 4,115 (3.5) | 45,920 (39.5) | 10,353 (8.9) |  | 2,764 (2.4) | 28,565 (24.6) | 6,998 (6.0) |
| **Female, n (%)** | 53,671 (46.2) | 480 (42.7) | 5,748 (44.1) | 1,242 (36.9) |  | 1,589 (38.6) | 21,154 (46.1) | 4,348 (42.0) |  | 1,186 (42.9) | 14,581 (51.0) | 3,343 (47.8) |
| **Age** | 42.6 (11.9) | 45.1 (14.2) | 42.3 (12.7) | 39.6 (12.4) |  | 45.7 (13.3) | 42.8 (12.1) | 40.8 (11.9) |  | 44.9 (11.7) | 42.8 (11.0) | 41.3 (10.5) |
| **Follow-up, years** | 4.6 (2.5) | 4.2 (2.3) | 4.1 (2.3) | 4.9 (2.5) |  | 4.3 (2.4) | 4.1 (2.4) | 4.8 (2.6) |  | 4.9 (2.5) | 5.0 (2.6) | 5.7 (2.6) |
| **Education, n (%)** |  |  |  |  |  |  |  |  |  |  |  |  |
| **No**  **schooling** | 2,497 (2.1) | 52 (4.6) | 349 (2.7) | 45 (1.3) |  | 102 (2.5) | 955 (2.1) | 152 (1.5) |  | 80 (2.9) | 649 (2.3) | 113 (1.6) |
| **Elementary** | 8,683 (7.5) | 149 (13.2) | 1,106 (8.5) | 199 (5.9) |  | 436 (10.6) | 3,368 (7.3) | 556 (5.4) |  | 268 (9.7) | 2,169 (7.6) | 432 (6.2) |
| **Junior High** | 5,019 (4.3) | 69 (6.1) | 651 (5.0) | 148 (4.4) |  | 175 (4.3) | 1,970 (4.3) | 401 (3.9) |  | 131 (4.7) | 1,192 (4.2) | 282 (4.0) |
| **Senior High** | 22,591 (19.4) | 226 (20.1) | 2,536 (19.5) | 680 (20.2) |  | 803 (19.5) | 8,519 (18.6) | 1,972 (19.0) |  | 550 (19.9) | 5,798 (20.3) | 1,507 (21.5) |
| **Vocational** | 26,964 (23.2) | 164 (14.6) | 2,865 (22.0) | 753 (22.4) |  | 812 (19.7) | 10,370 (22.6) | 2,425 (23.4) |  | 613 (22.2) | 7,066 (24.7) | 1,896 (27.1) |
| **College/**  **University** | 34,646 (29.8) | 306 (27.2) | 3,818 (29.3) | 1,061 (31.5) |  | 1,140 (27.7) | 14,142 (30.8) | 3,361 (32.5) |  | 729 (26.4) | 8,138 (28.5) | 1,951 (27.9) |
| **Graduate**  **School** | 15,828 (13.6) | 159 (14.1) | 1,697 (13.0) | 480 (14.3) |  | 647 (15.7) | 6,596 (14.4) | 1,486 (14.4) |  | 393 (14.2) | 3,553 (12.4) | 817 (11.7) |
| **Smoking status, n (%)** |  |  |  |  |  |  |  |  |  |  |  |  |
| **Never** | 82,030 (70.6) | 814 (72.3) | 9,221 (70.8) | 2,326 (69.1) |  | 2,827 (68.7) | 32,702 (71.2) | 7,259 (70.1) |  | 1,929 (69.8) | 20,076 (70.3) | 4,876 (69.7) |
| **2^nd^ hand** | 4,373 (3.8) | 42 (3.7) | 416 (3.2) | 101 (3.0) |  | 181 (4.4) | 1,660 (3.6) | 405 (3.9) |  | 128 (4.6) | 1,115 (3.9) | 325 (4.6) |
| **Former** | 6,981 (6.0) | 43 (3.9) | 762 (5.9) | 195 (5.8) |  | 256 (6.2) | 2,666 (5.8) | 619 (6.0) |  | 174 (6.3) | 1,791 6.3) | 475 (6.8) |
| **Frequently** | 4,536 (3.9) | 49 (4.3) | 503 (3.9) | 149 (4.4) |  | 173 (4.2) | 1,792 (3.9) | 402 (3.9) |  | 122 (4.4) | 1,086 (3.8) | 260 (3.8) |
| **Daily** | 18,308 (15.8) | 177 (15.7) | 2,120 (16.3) | 595 (17.7) |  | 679 (16.5) | 7,100 (15.5) | 1,668 (16.1) |  | 411(14.9) | 4,496 (15.7) | 1,062 (15.2) |
| **Alcohol consumption, n (%)** |  |  |  |  |  |  |  |  |  |  |  |  |
| **None** | 95,693 (82.3) | 925 (82.2) | 10,642 (81.7) | 2,717 (80.7) |  | 3,361 (81.7) | 37,669 (82.0) | 8,413 (81.3) |  | 2,320 (83.9) | 23,882 (83.6) | 5,764 (82.4) |
| **Former** | 2,473 (2.1) | 32 (2.8) | 310 (2.4) | 66 (2.0) |  | 113 (2.7) | 933 (2.0) | 216 (2.1) |  | 71 (2.6) | 568 (2.0) | 164 (2.3) |
| **1-2 times/wk** | 12,113 (10.4) | 106 (9.4) | 1,393 (10.7) | 401 (11.9) |  | 436 (10.6) | 4,901 (10.7) | 1,165 (11.3) |  | 213 (7.7) | 2,769 (9.7) | 729 (10.4) |
| **3-4 times/wk** | 4,035 (3.5) | 39 (3.5) | 451 (3.5) | 119 (3.5) |  | 121 (2.9) | 1,661 (3.6) | 362 (3.5) |  | 108 (3.9) | 918 (3.2) | 256 (3.7) |
| **Daily** | 1,914 (1.6) | 23 (2.0) | 226 (1.7) | 63 (1.9) |  | 84 (2.0) | 756 (1.6) | 197 (1.9) |  | 52 (1.9) | 428 (1.5) | 85 (1.2) |
| **Diet^1^** | 2.2 (1.2) | 2.2 (1.3) | 2.1 (1.2) | 2.0 (1.2) |  | 2.3 (1.3) | 2.2 (1.2) | 2.1 (1.2) |  | 2.3 (1.2) | 2.2 (1.2) | 2.1 (1.2) |
| **Sleep, n (%)^2^** |  |  |  |  |  |  |  |  |  |  |  |  |
| **≤4hrs** | 930 (0.8) | 14 (1.2) | 150 (1.2) | 42 (1.2) |  | 26 (0.6) | 343 (0.7) | 75 (0.7) |  | 29 (1.0) | 206 (0.7) | 45 (0.6) |
| **>4 to ≤6hrs** | 25,218 (21.7) | 292 (26.0) | 2,876 (22.1) | 815 (24.2) |  | 948 (23.0) | 9,920 (21.6) | 2,317 (22.4) |  | 607 (22.0) | 5,982 (20.9) | 1,461 (20.9) |
| **>6 to ≤8hrs** | 82,570 (71.0) | 745 (66.2) | 9,156 (70.3) | 2,291 (68.1) |  | 2,876 (69.9) | 32,772 (71.4) | 7,298 (70.5) |  | 1,963 (71.0) | 20,415 (71.5) | 5,054 (72.2) |
| **>8hrs** | 7,510 (6.5) | 74 (6.6) | 840 (6.5) | 218 (6.5) |  | 265 (6.4) | 2,885 (6.3) | 663 (6.4) |  | 165 (6.0) | 1,962 (6.9) | 438 (6.3) |
| **Hypertension, n (%)** | 9,743 (8.4) | 127 (11.3) | 1,165 (8.9) | 195 (5.8) |  | 473 (11.5) | 4,030 (8.8) | 663 (6.4) |  | 283 (10.2) | 2,380 (8.3) | 427 (6.1) |
| **Diabetes, n (%)** | 3,292 (2.8) | 66 (5.9) | 392 (3.0) | 50 (1.5) |  | 243 (5.9) | 1,304 (2.8) | 160 (1.5) |  | 180 (6.5) | 806 (2.8) | 91 (1.3) |

^1^Fruits and vegetable servings/day

^2^hours/day

Supplemental Table 3: Physical activity and adiposity change values between baseline and follow-up assessments

Physical activity (MET-hrs/week)

| Inactive | | Insufficient | | | Sufficient | |
| --- | --- | --- | --- | --- | --- | --- |
| Stable | Increased | Decreased | Stable | Increased | Decreased | Stable |
| 0.0 [0.0, 0.0] | 2.0 [1.3, 3.9] | -1.7 [-3.8, -1.3] | 0.0 [-1.5, 0.1] | 4.0 [2.0, 5.5] | -6.0 [-9.3, -3.0] | 0.0 [-3.0, 5.5] |

Body mass index (kg/m^2^)

| Healthy | | Overweight | | | Obese | |
| --- | --- | --- | --- | --- | --- | --- |
| Stable | Increased | Decreased | Stable | Increased | Decreased | Stable |
| 0.3 [-0.3, 0.9] | 1.5 [0.7, 2.5] | -1.2 [-1.9, -0.8] | 0.1 [-0.3, 0.6] | 1.6 [1.0, 2.3] | -1.5 [-2.5, -0.9] | 0.4 [-0.3, 1.2] |

Body fat (%)

| Low | | Moderate | | | High | |
| --- | --- | --- | --- | --- | --- | --- |
| Stable | Increased | Decreased | Stable | Increased | Decreased | Stable |
| 0.5 [-1.1, 1.2] | 4.6 [2.8, 6.7] | -3.0 [-4.4, -1.9] | 0.1 [-0.7, 1.1] | 3.6 [2.3, 5.0] | -3.8 [-5.8, -2.3] | 0.5 [-1.3, 2.4] |

Waist circumference (cm)

| Men | | | | | | |
| --- | --- | --- | --- | --- | --- | --- |
| Low | | Moderate | | | High | |
| Stable | Increased | Decreased | Stable | Increased | Decreased | Stable |
| 1.1 [-1.2, 3.2] | 7.4 [4.1, 10.0] | -5.4 [-7.3, -3.8] | 1.3 [-1.3, 2.7] | 6.2 [4.5, 8.0] | -5.7 [-8.3, -3.9] | 1.4 [-2.6, 4.2] |

| Women | | | | | | |
| --- | --- | --- | --- | --- | --- | --- |
| Low | | Moderate | | | High | |
| Stable | Increased | Decreased | Stable | Increased | Decreased | Stable |
| 0.4 [-1.2, 2.4] | 5.3 [2.7, 8.3] | -4.0 [-6.0, -2.4] | 1.7 [-1.5, 2.3] | 6.3 [4.7, 9.1] | -4.8 [-7.7, -3.4] | 0.6 [-1.3, 3.2] |


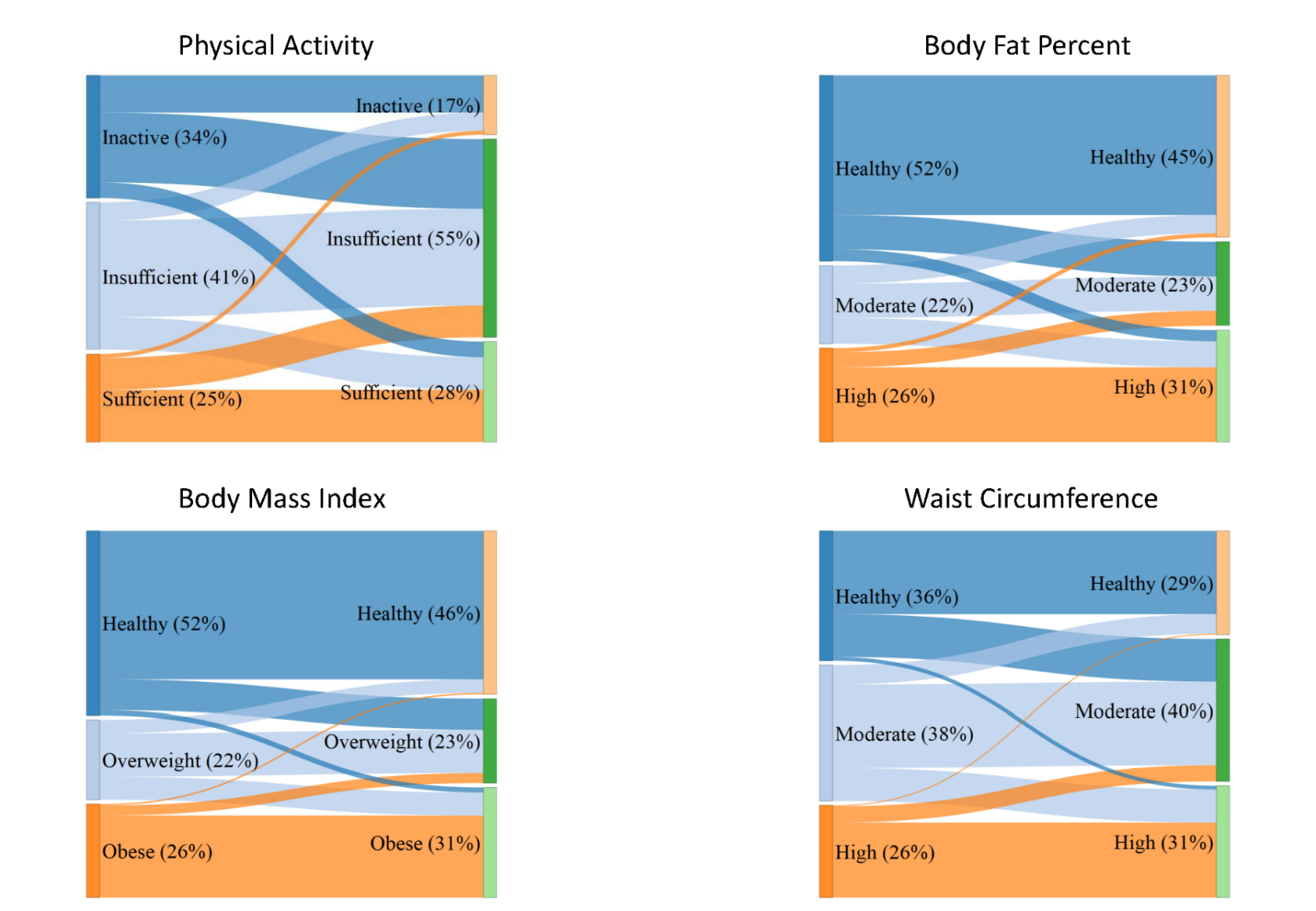
Supplemental Figure 2: Degree of physical activity, body fat percent, body mass index, and waist circumference changes between baseline and follow up assessments

Left side indicates status at baseline assessment, right side indicates status at follow up assessment. Width of cross-over bars indicates proportion of participants from each baseline group

Supplemental Table 4: Characteristics of participants who were excluded from analyses

|  | Baseline* | Follow-up* |
| --- | --- | --- |
| **Total** | 46,248 | 46,248 |
| **Female, n (%)** | 28,428 (61.2) | 28,428 (61.2) |
| **Age** | 40.2 (15.0) | 45.1 (15.3) |
| **Education, n (%)** |  |  |
| **No**  **schooling** | 1,738 (3.9) | 1,605 (3.6) |
| **Elementary** | 7,087 (15.8) | 6,208 (13.9) |
| **Junior High** | 3,926 (8.8) | 3,647 (8.2) |
| **Senior High** | 10,713 (23.9) | 10,259 (23.0) |
| **Vocational** | 9,353 (20.9) | 8,910 (20.0) |
| **College/**  **University** | 9,234 (20.6) | 10,406 (23.3) |
| **Graduate**  **School** | 2,743 (6.1) | 3,541 (7.9) |
| **Smoking status, n (%)** |  |  |
| **Never** | 31,348 (71.8) | 29,869 (71.5) |
| **2^nd^ hand** | 2,799 (6.4) | 3,462 (8.3) |
| **Former** | 2,413 (5.5) | 3,297 (7.9) |
| **Frequently** | 1,255 (2.9) | 1,737 (4.2) |
| **Daily** | 5,825 (13.3) | 3,428 (8.2) |
| **Alcohol consumption, n (%)** |  |  |
| **None** | 33,616 (84.8) | 33,807 (84.9) |
| **Former** | 1,214 (3.1) | 1,217 (3.1) |
| **1-2 times/wk** | 3,001 (7.6) | 3,008 (7.6) |
| **3-4 times/wk** | 1,122 (2.8) | 1,104 (2.8) |
| **Daily** | 704 (1.8) | 666 (1.7) |
| **Diet^1^** | 2.0 (1.2) | 2.2 (1.2) |
| **Sleep, n (%)^2^** |  |  |
| **≤4hrs** | 529 (1.2) | 535 (1.3) |
| **>4 to ≤6hrs** | 8,441 (19.2) | 9,377 (22.3) |
| **>6 to ≤8hrs** | 30,235 (68.6) | 28,443 (67.5) |
| **>8hrs** | 4,858 (11.0) | 3,782 (9.0) |
| **Hypertension, n (%)** | 4,054 (8.7) | 6,403 (13.8) |
| **Diabetes, n (%)** | 1,185 (2.6) | 2,130 (4.6) |
| **Physical**  **Activity** | 2.9 [0.0, 8.8] | 1.9 [0.0, 8.8] |
| **Body mass**  **Index** | 21.3 [18.1, 24.4] | 21.8 [18.9, 24.8] |
| **Body fat**  **percent** | 23.1 [19.3, 28.8] | 24.6 [20.6, 29.9] |
| **Waist**  **Circumference** | 71.0 [64.0, 81.0] | 73.0 [66.0, 83.0] |

*Due to missing data, values may not add up to total sample size

Supplemental Table 5: Baseline characteristics of participants who had a re-examination assessment and participants who only had a baseline assessment

|  | **Participants with re-examination assessment*** | **Participants with only a baseline assessment*** |
| --- | --- | --- |
| **n** | 162,476 | 280,898 |
| **Physical activity (median [IQR])** | 2.1 [0.0, 7.9] | 2.2 [0.0, 7.9] |
| **Body mass index (median [IQR])** | 22.5 [20.3, 24.9] | 22.7 [20.1, 25.2] |
| **Body fat percent (median [IQR])** | 25.0 [21.1, 29.5] | 25.3 [21.4, 29.6] |
| **Waist circumference (median [IQR])** | 75.0 [68.0, 83.0] | 75.0 [67.7, 83.4] |
| **Age** | 38.6 (12.8) | 39.6 (14.2) |
| **Female, n (%)** | 82,021 (50.5) | 160,276 (57.1) |
| **Education, n (%)** |  |  |
| **No**  **schooling** | 4,205 (2.6) | 9,272 (3.4) |
| **Elementary** | 16,286 (10.1) | 37,126 (13.6) |
| **Junior High** | 9,566 (5.9) | 20,981 (7.7) |
| **Senior High** | 35,205 (21.9) | 63,284 (23.1) |
| **Vocational** | 37,864 (23.5) | 59,933 (21.9) |
| **College/**  **University** | 41,900 (26.1) | 62,385 (22.8) |
| **Graduate**  **School** | 15,799 (9.8) | 20,718 (7.6) |
| **Smoking status, n (%)** |  |  |
| **Never** | 110,363 (69.1) | 190,012 (70.7) |
| **2^nd^ hand** | 9,185 (5.8) | 16,518 (6.1) |
| **Former** | 9,364 (5.9) | 15,189 (5.7) |
| **Frequently** | 5,631 (3.5) | 8,479 (3.2) |
| **Daily** | 25,128 (15.7) | 38,424 (14.3) |
| **Alcohol consumption, n (%)** |  |  |
| **None** | 129,699 (83.3) | 211,480 (84.2) |
| **Former** | 3,692 (2.4) | 6,979 (2.8) |
| **1-2 times/wk** | 14,853 (9.5) | 21,040 (8.4) |
| **3-4 times/wk** | 4,998 (3.2) | 7,502 (3.0) |
| **Daily** | 2,446 (1.6) | 4,234 (1.7) |
| **Diet^1^** | 2.0 (1.2) | 2.0 (1.2) |
| **Sleep, n (%)^2^** |  |  |
| **≤4hrs** | 1,421 (0.9) | 2,895 (1.1) |
| **>4 to ≤6hrs** | 30,389 (19.0) | 51,625 (19.1) |
| **>6 to ≤8hrs** | 114,810 (71.7) | 188,967 (69.9) |
| **>8hrs** | 13,474 (8.4) | 27,044 (10.0) |

*Due to missing data, values may not add up to total sample size


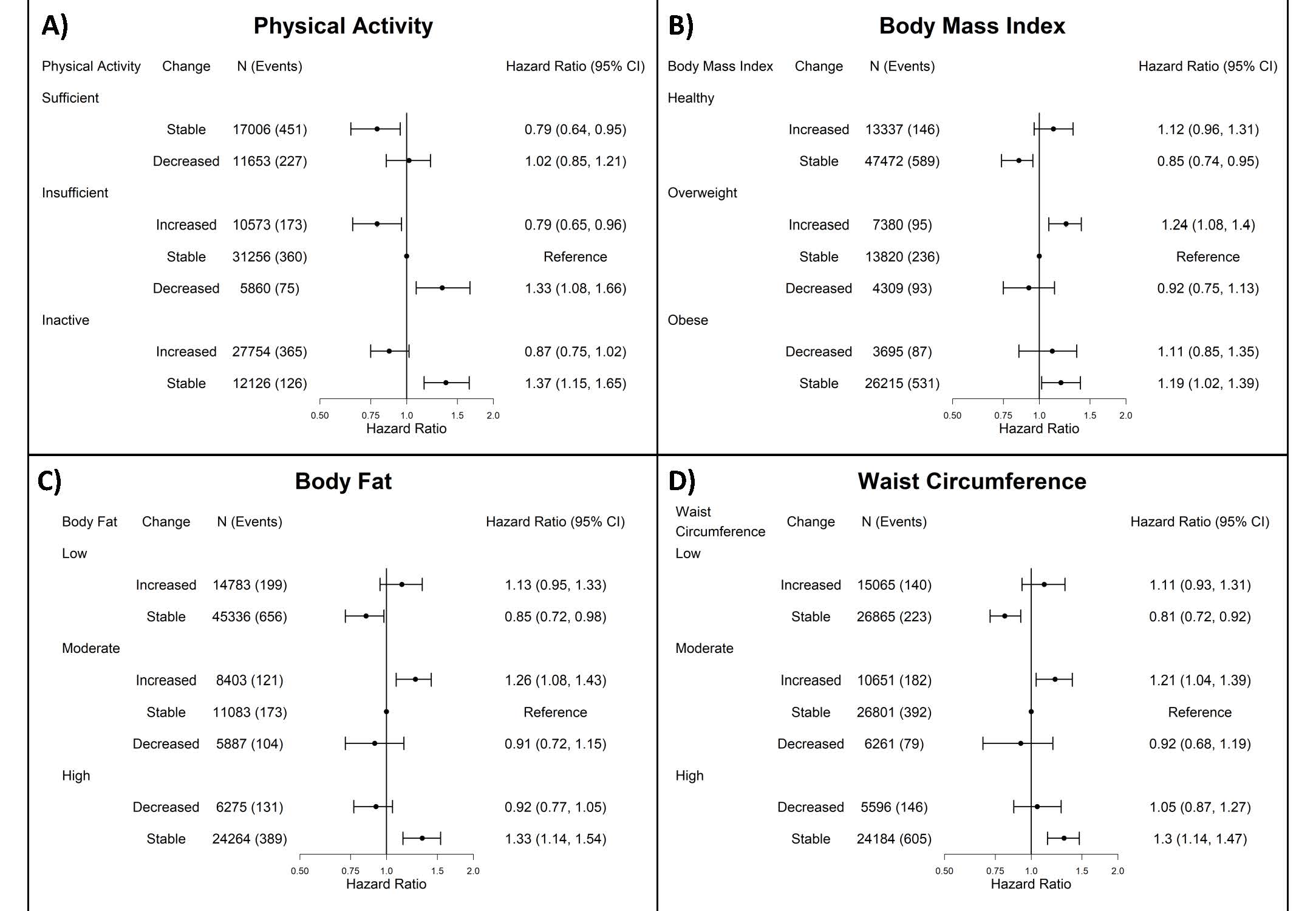
Supplemental Figure 3: Independent association for physical activity, body fat, body mass index, and waist circumference changes with cancer mortality (physical activity: n=116,228, events=1,777; body fat percent: n=116,031, events=1,773; body mass index: n=116,228, events=1,777; waist circumference: n=3,816, events=1,767)

All results are adjusted for: age, sex, physical activity group (for adiposity exposures), body mass index group (for physical activity as the exposure), smoking status, alcohol consumption, sleep duration, diet (fruits and vegetables consumption), and education


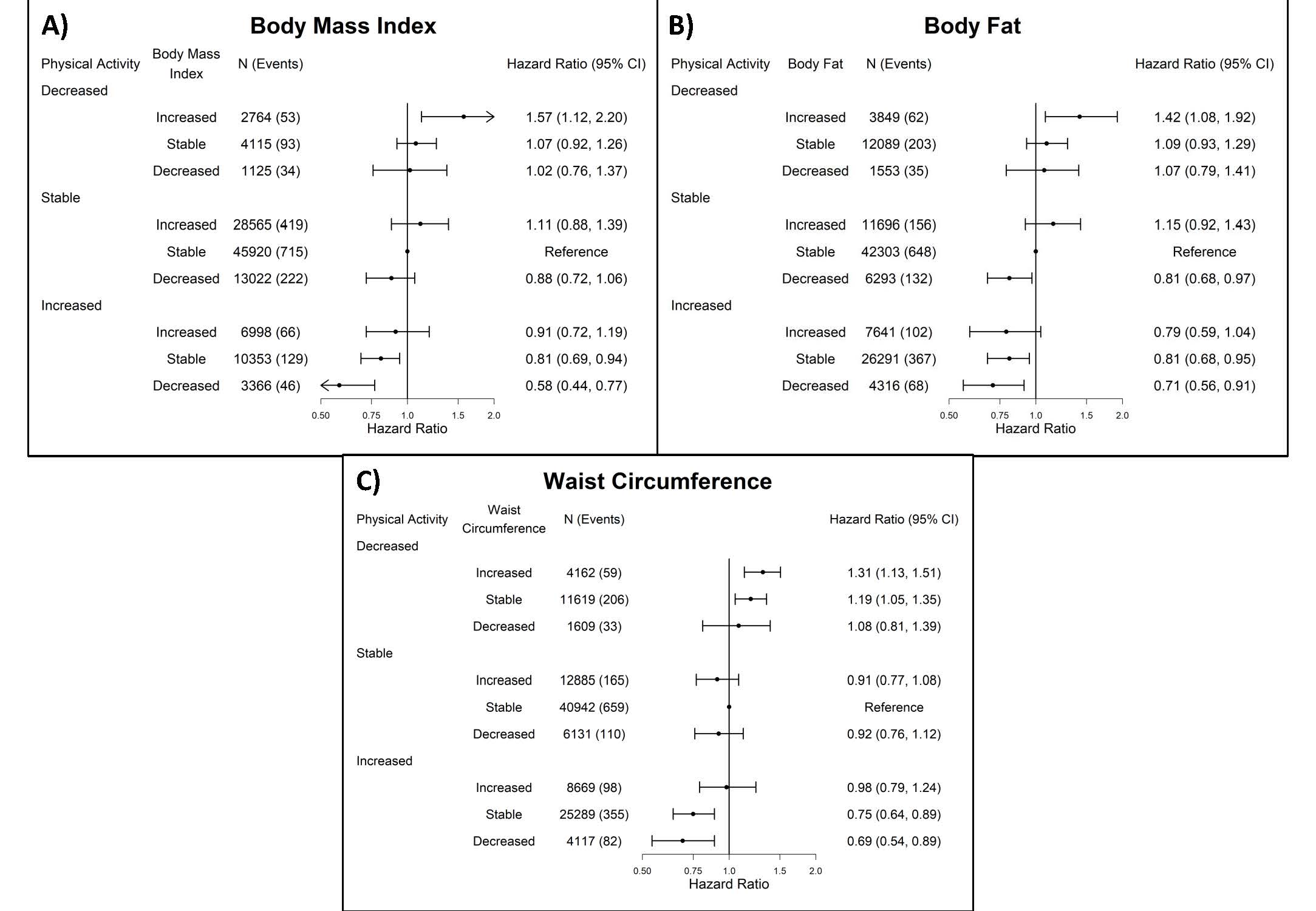
Supplemental Figure 4: Joint association of physical activity and adiposity changes with cancer mortality (body fat: n= 116,031, events= 1,773; body mass index: n=116,228, events= 1,777; waist circumference: n= 115,423; events= 1,767)

All results are adjusted for: age, sex, baseline physical activity, baseline adiposity (body fat percentage, body mass index, or waist circumference), smoking status, alcohol consumption, sleep duration, diet (fruits and vegetables consumption), and education

Supplemental Table 5: Independent physical activity change E-values for all-cause, cardiovascular disease, and cancer mortality

| Baseline | Change | All-cause mortality | Cardiovascular disease mortality | Cancer mortality |
| --- | --- | --- | --- | --- |
| Sufficient |  |  |  |  |
|  | Stable | 1.80 (1.39) | 2.10 (1.46) | 1.84 (1.37) |
|  | Decreased | 1.49 (1.00) | 1.64 (1.00) | 1.16 (1.00) |
| Insufficient |  |  |  |  |
|  | Increased | 1.63 (1.25) | 2.03 (1.29) | 1.81 (1.23) |
|  | Stable | Reference | Reference | Reference |
|  | Decreased | 2.13 (1.64) | 2.91 (1.69) | 1.96 (1.33) |
| Inactive |  |  |  |  |
|  | Increased | 1.63 (1.29) | 1.85 (1.31) | 1.53 (1.00) |
|  | Stable | 2.28 (1.83) | 2.62 (1.53) | 2.04 (1.51) |

Supplemental Table 6: Independent adiposity change E-values for all-cause, cardiovascular disease, and cancer mortality

| Baseline | Change | All-cause mortality | | | Cardiovascular disease mortality | | | Cancer mortality | | |
| --- | --- | --- | --- | --- | --- | --- | --- | --- | --- | --- |
|  |  | BF% | BMI | WC | BF% | BMI | WC | BF% | BMI | WC |
| Healthy/  Low |  |  |  |  |  |  |  |  |  |  |
|  | Increased | 1.26 (1.00) | 1.37 (1.00) | 1.35 (1.00) | 1.68 (1.00) | 1.22 (1.00) | 1.49 (1.00) | 1.46 (1.00) | 1.52 (1.00) | 1.55 (1.00) |
|  | Stable | 1.82 (1.30) | 1.74 (1.50) | 1.77 (1.33) | 1.96 (1.34) | 1.93 (1.46) | 1.85 (1.27) | 1.65 (1.21) | 1.68 (1.32) | 1.70 (1.32) |
| Overweight/Moderate |  |  |  |  |  |  |  |  |  |  |
|  | Increased | 1.81 (1.35) | 1.79 (1.31) | 1.80 (1.30) | 2.01 (1.50) | 2.07 (1.46) | 1.87 (1.35) | 1.92 (1.45) | 1.77 (1.00) | 1.72 (1.31) |
|  | Stable | Reference | | | Reference | | | Reference | | |
|  | Decreased | 1.55 (1.0) | 1.43 (1.00) | 1.44 (1.00) | 1.72 (1.00) | 1.33 (1.00) | 1.66 (1.00) | 1.39 (1.00) | 1.36 (1.00) | 1.42 (1.00) |
| Obese/  High |  |  |  |  |  |  |  |  |  |  |
|  | Stable | 2.13 (1.67) | 2.00 (1.49) | 1.98 (1.46) | 2.57 (1.61) | 2.12 (1.34) | 2.45 (1.77) | 1.37 (1.00) | 1.53 (1.00) | 1.31 (1.00) |
|  | Decreased | 1.46 (1.00) | 1.43 (1.00) | 1.34 (1.00) | 1.51 (1.00) | 1.37 (1.00) | 1.46 (1.00) | 2.05 (1.64) | 1.74 (1.28) | 1.99 (1.58) |

Supplemental Table 7: Joint physical activity and adiposity change E-values for all-cause, cardiovascular disease, and cancer mortality

| Physical Activity | Adiposity | All-cause mortality | | | Cardiovascular disease mortality | | | Cancer mortality | | |
| --- | --- | --- | --- | --- | --- | --- | --- | --- | --- | --- |
|  |  | BF% | BMI | WC | BF% | BMI | WC | BF% | BMI | WC |
| Decreased |  |  |  |  |  |  |  |  |  |  |
|  | Increased | 2.14 (1.54) | 2.72 (1.99) | 2.45 (2.02) | 2.54 (1.54) | 2.47 (1.57) | 2.24 (1.28) | 2.10 (1.24) | 2.51 (1.45) | 1.95 (1.46) |
|  | Stable | 1.70 (1.40) | 1.69 (1.38) | 1.65 (1.38) | 2.08 (1.34) | 1.80 (1.32) | 1.77 (1.00) | 1.37 (1.00) | 1.39 (1.00) | 1.62 (1.24) |
|  | Decreased | 1.43 (1.00) | 1.34 (1.00) | 1.34 (1.00) | 1.76 (1.00) | 1.50 (1.00) | 1.32 (1.00) | 1.32 (1.00) | 1.16 (1.00) | 1.38 (1.00) |
| Stable |  |  |  |  |  |  |  |  |  |  |
|  | Increased | 1.65 (1.18) | 1.11 (1.00) | 1.11(1.00) | 1.28 (1.00) | 1.46 (1.00) | 1.28 (1.00) | 1.55 (1.00) | 1.46 (1.00) | 1.43 (1.00) |
|  | Stable | Reference | | | Reference | | | Reference | | |
|  | Decreased | 1.53 (1.11) | 1.45 (1.00) | 1.92 (1.51) | 1.16 (1.00) | 1.16 (1.00) | 1.56 (1.00) | 1.81 (1.25) | 1.56 (1.00) | 1.39 (1.00) |
| Increased |  |  |  |  |  |  |  |  |  |  |
|  | Increased | 1.77 (1.21) | 1.51 (1.00) | 1.70 (1.11) | 2.61 (1.24) | 1.78 (1.00) | 2.50 (1.00) | 1.81 (1.00) | 1.43 (1.00) | 1.16 (1.00) |
|  | Stable | 2.00 (1.67) | 1.96 (1.63) | 2.08 (1.77) | 2.64 (1.70) | 2.61 (1.74) | 2.80 (1.87) | 1.77 (1.34) | 1.74 (1.27) | 1.92 (1.43) |
|  | Decreased | 2.29 (1.79) | 2.84 (2.21) | 2.75 (2.22) | 2.94 (1.45) | 3.87 (1.92) | 3.33 (1.67) | 2.08 (1.36) | 2.72 (1.85) | 2.17 (1.43) |


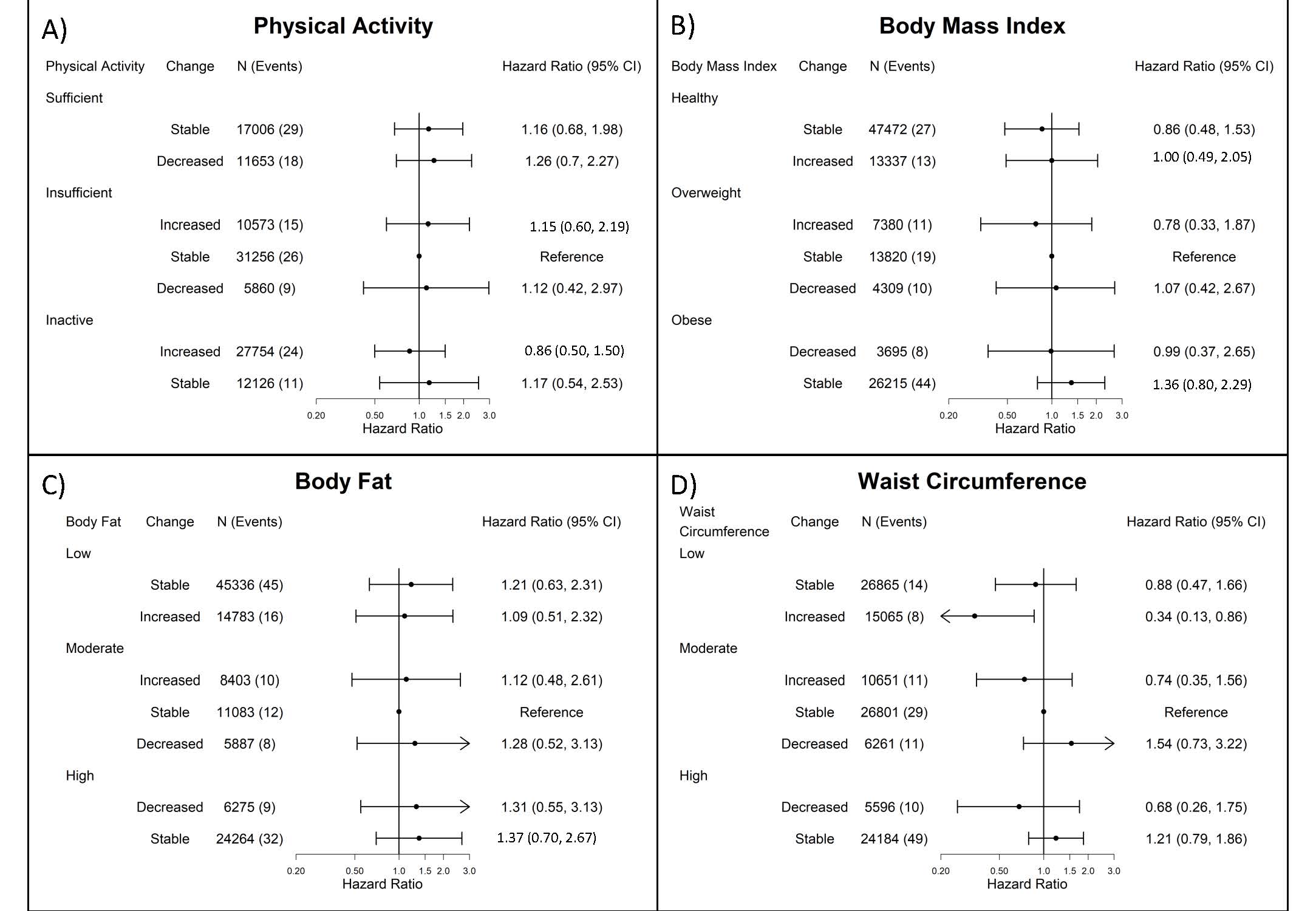
Supplemental Figure 5: Independent association for physical activity, body fat percent, body mass index, and waist circumference with negative control outcome of accidents/sequelae of transport or other accidents (physical activity: n=116,228, events=132; body fat percent: n=116,031, events=132; body mass index: n=116,228, events=132; waist circumference: n=3,816, events=132)

All results are adjusted for: age, sex, physical activity group (for adiposity exposures), body mass index group (for physical activity as the exposure), smoking status, alcohol consumption, sleep duration, diet (fruits and vegetables consumption), and education


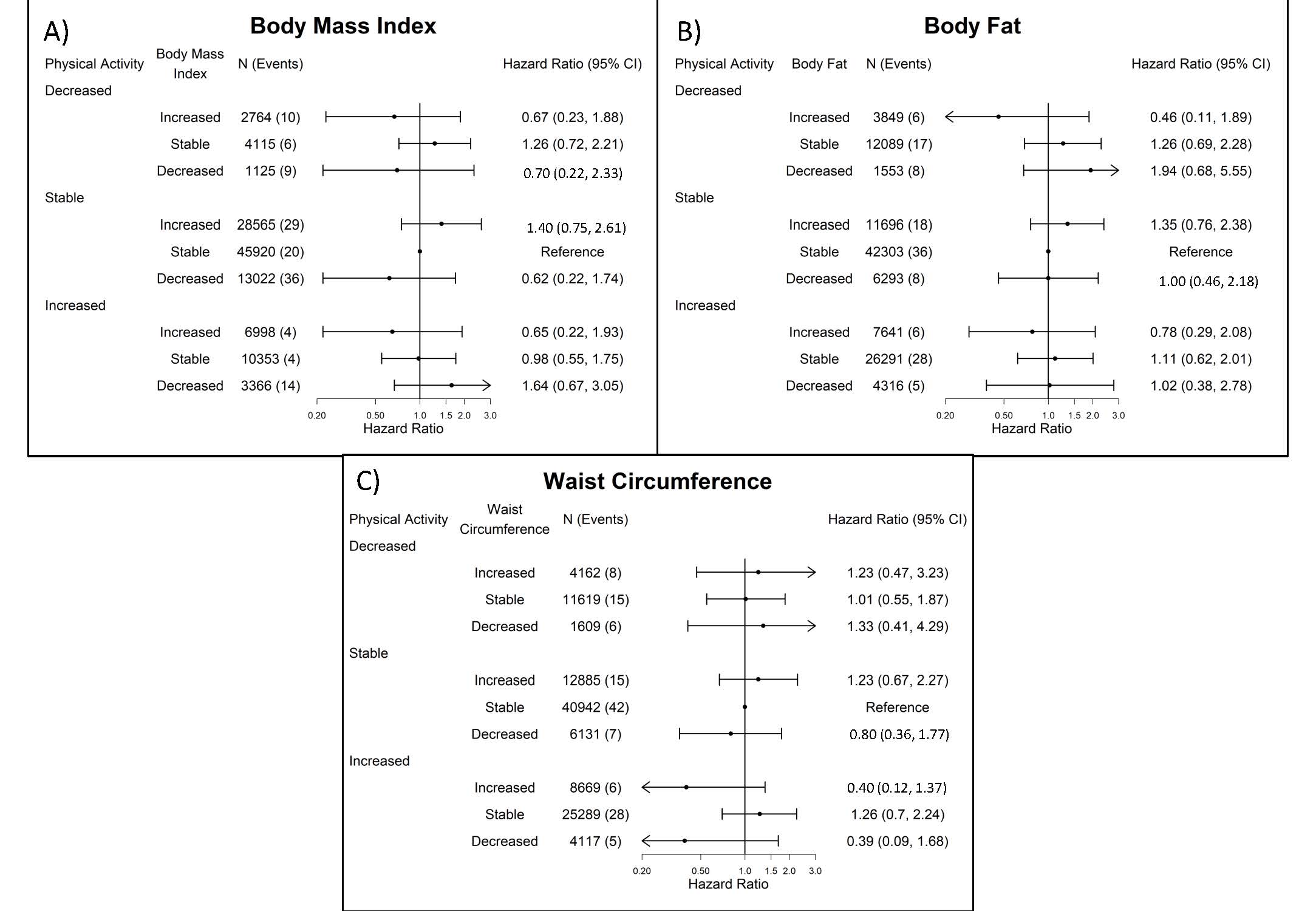
Supplemental Figure 6: Joint association of physical activity and adiposity changes with negative control outcome of accidents/sequelae of transport or other accidents (body fat: n= 116,031, events= 132; body mass index: n=116,228, events= 132; waist circumference: n= 115,423; events= 132)

All results are adjusted for: age, sex, baseline physical activity, baseline adiposity (body fat percentage, body mass index, or waist circumference), smoking status, alcohol consumption, sleep duration, diet (fruits and vegetables consumption), and education


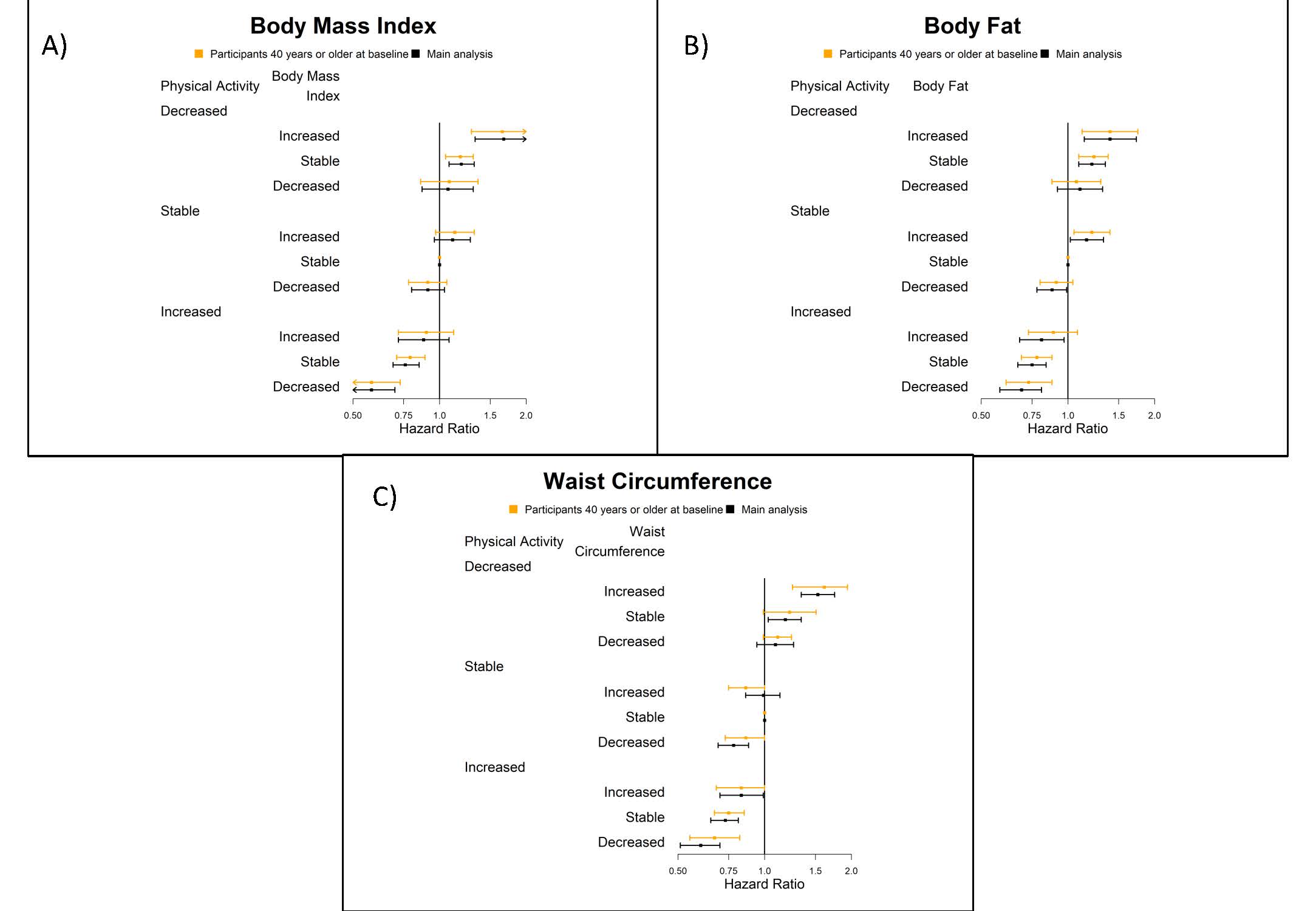
Supplemental Figure 7: Joint association of physical activity and adiposity changes with all-cause mortality among participants 40 years or older at baseline assessment (body mass index: n=43,107, events=3,165; body fat: n=43,038, events=3,160; waist circumference: n=42,881, events=3,150)

All results are adjusted for: age, sex, baseline physical activity, baseline adiposity (body fat percentage, body mass index, or waist circumference), smoking status, alcohol consumption, sleep duration, diet (fruits and vegetables consumption), and education


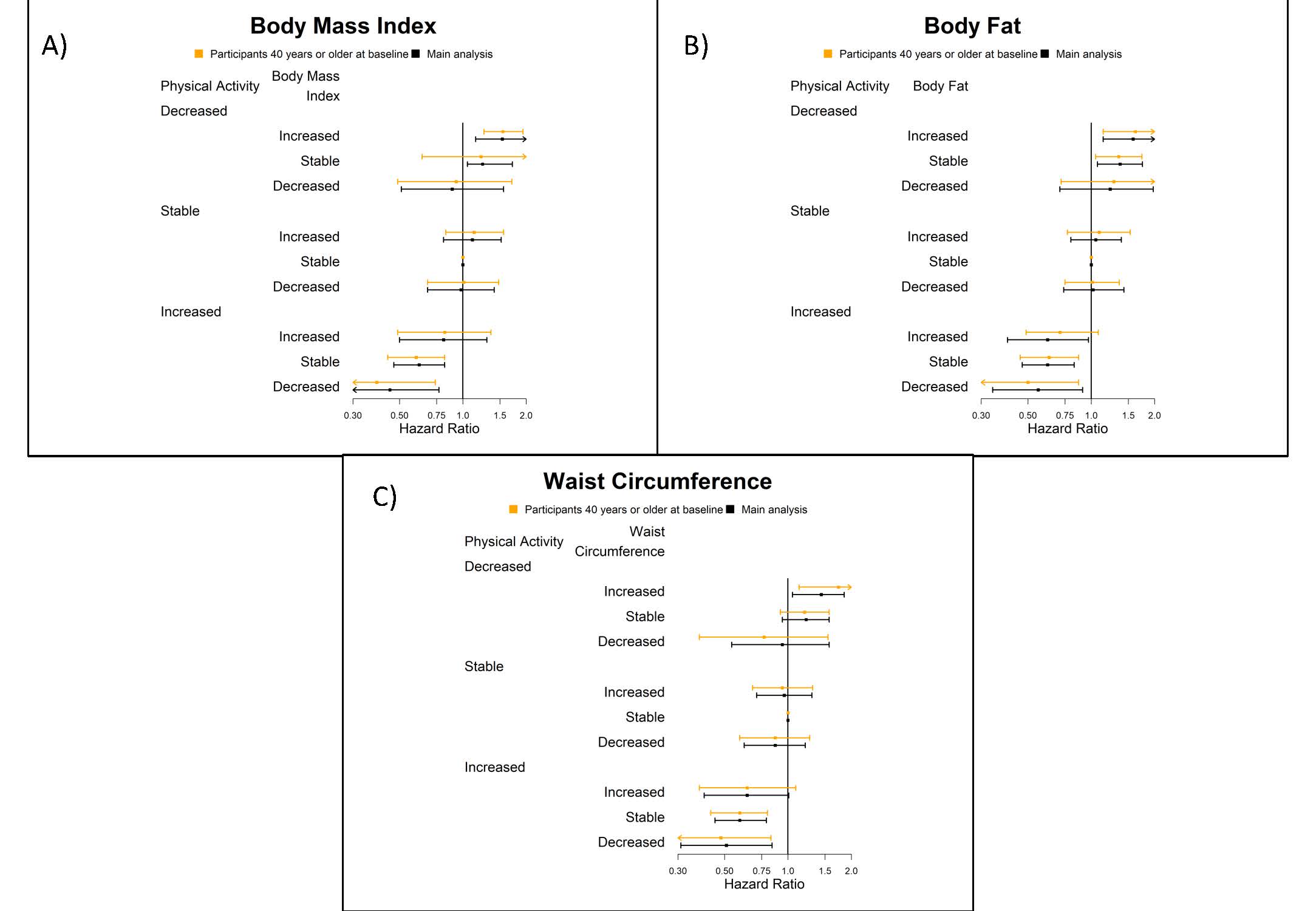
Supplemental Figure 8: Joint association of physical activity and adiposity changes with cardiovascular disease mortality among participants 40 years or older at baseline assessment (body mass index: n=43,107, events=529; body fat: n=43,038, events=528; waist circumference: n=42,881, events=528)

All results are adjusted for: age, sex, baseline physical activity, baseline adiposity (body fat percentage, body mass index, or waist circumference), smoking status, alcohol consumption, sleep duration, diet (fruits and vegetables consumption), and education


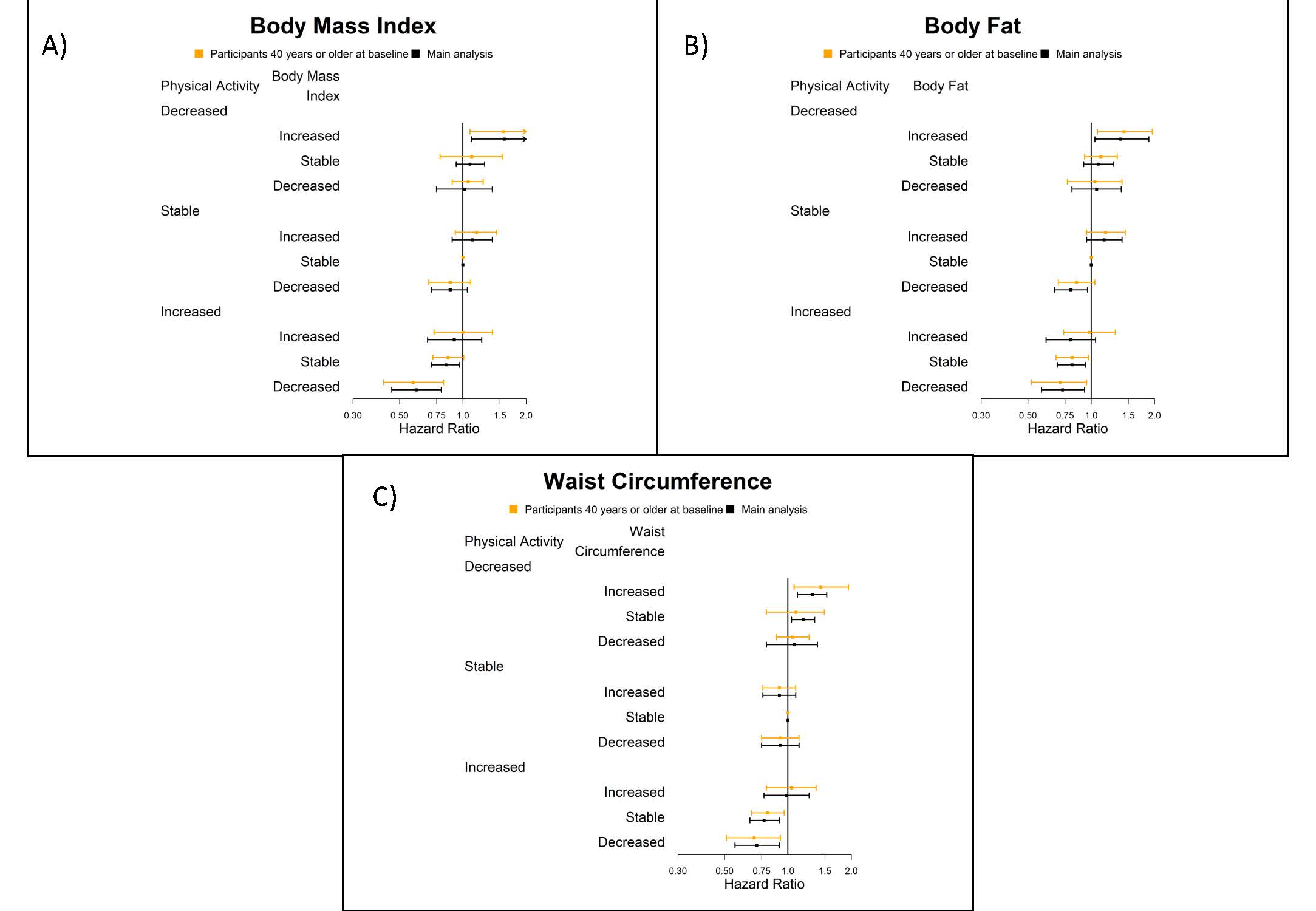
Supplemental Figure 9: Joint association of physical activity and adiposity changes with cancer mortality among participants 40 years or older at baseline assessment (body mass index: n=43,107, events=1,429; body fat: n=43,038, events=1,428; waist circumference: n=42,881, events=1,423)

All results are adjusted for: age, sex, baseline physical activity, baseline adiposity (body fat percentage, body mass index, or waist circumference), smoking status, alcohol consumption, sleep duration, diet (fruits and vegetables consumption), and education


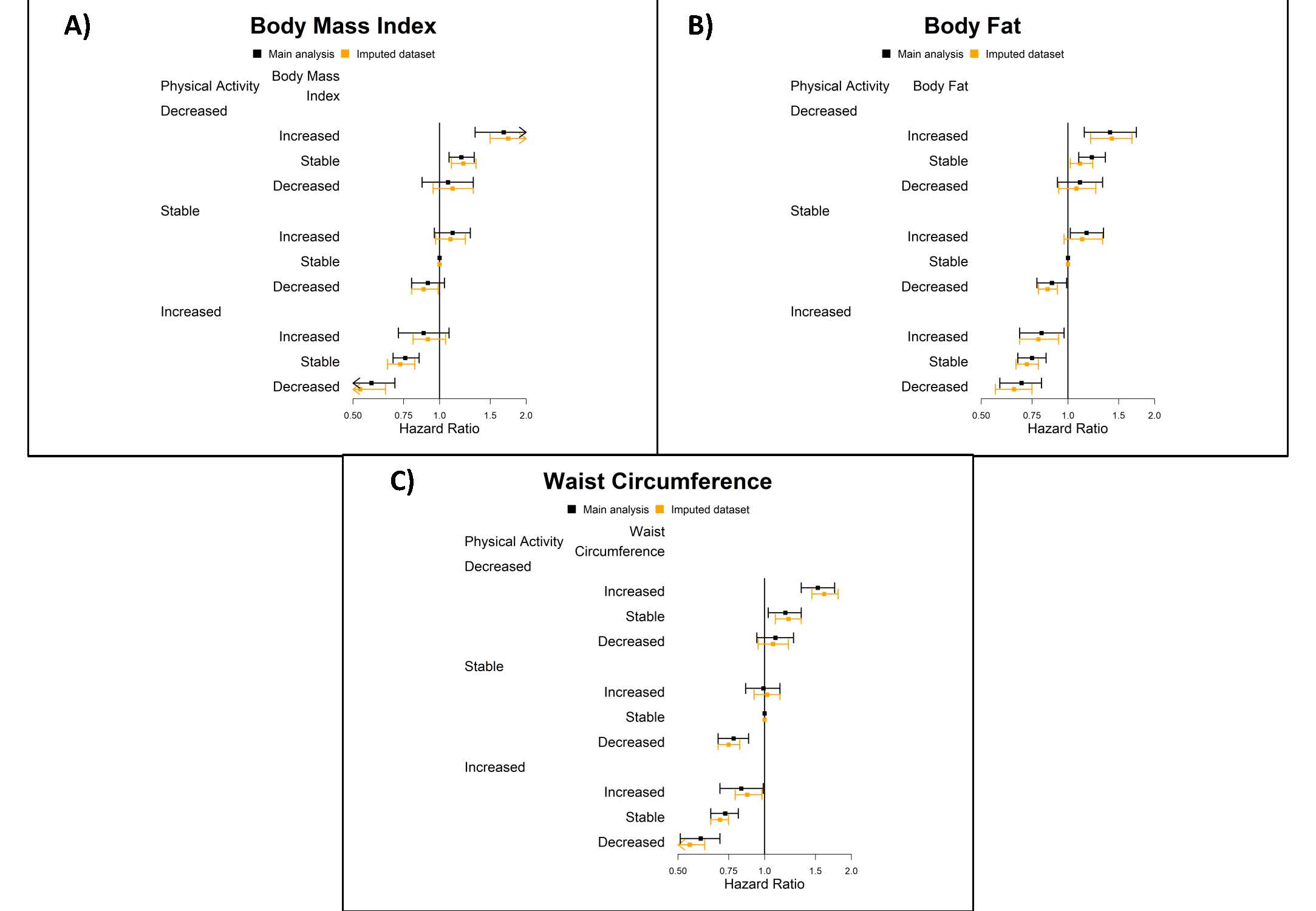
Supplemental Figure 10: Joint association of physical activity and adiposity changes with all-cause mortality using non-imputed and imputed datasets

Adjusted for: age, sex, baseline physical activity, baseline adiposity (body fat percentage, body mass index, or waist circumference), smoking status, alcohol consumption, sleep duration, diet (fruits and vegetables consumption), and education. Imputed dataset is adjusted for prevalent CVD and cancer.


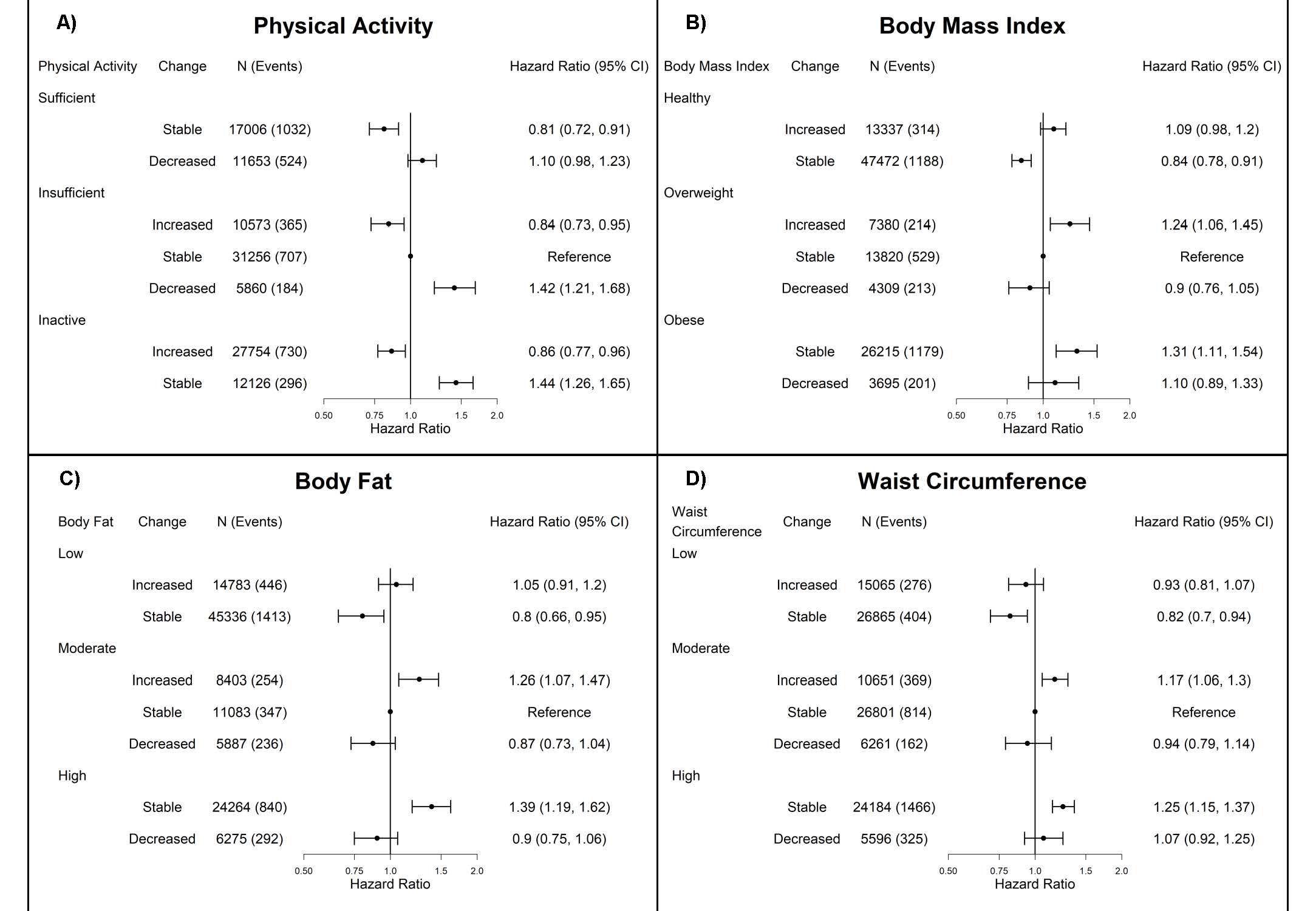
Supplemental Figure 11: Independent association for physical activity, body fat percent, body mass index, and waist circumference changes with all-cause mortality with adjustment for hypertension and diabetes, and mutual adjustment for physical activity and adiposity.

Adjusted for: age, sex, physical activity group (for adiposity exposures), body mass index group (for physical activity as the exposure), smoking status, alcohol consumption, sleep duration, diet (fruits and vegetables consumption), education, diabetes, and hypertension


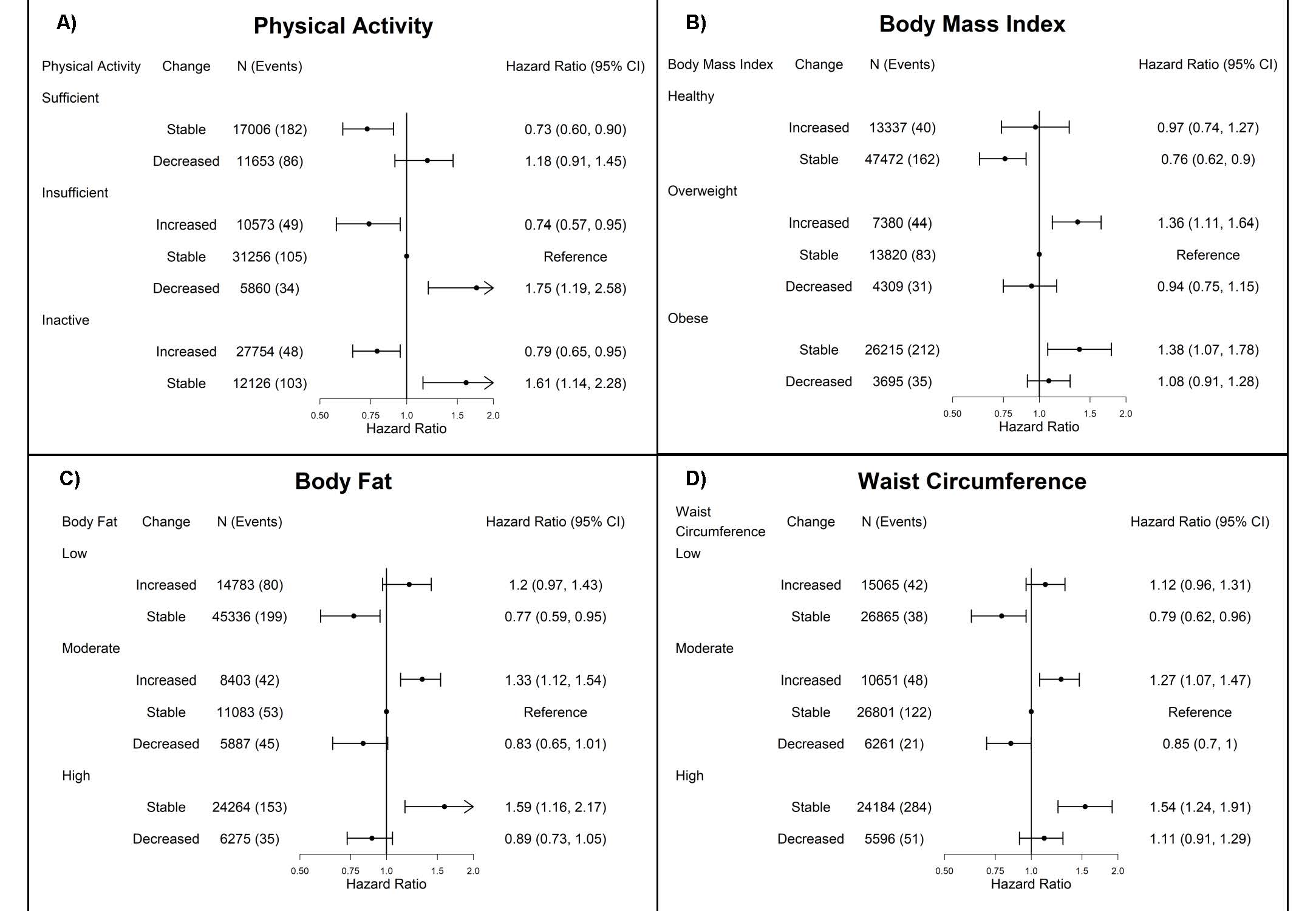
Supplemental Figure 12: Independent association for physical activity, body fat, body mass index, and waist circumference changes with CVD mortality with adjustment for hypertension and diabetes, and mutual adjustment for physical activity and adiposity.

Adjusted for: age, sex, physical activity group (for adiposity exposures), body mass index group (for physical activity as the exposure), smoking status, alcohol consumption, sleep duration, diet (fruits and vegetables consumption), education, diabetes, and hypertension


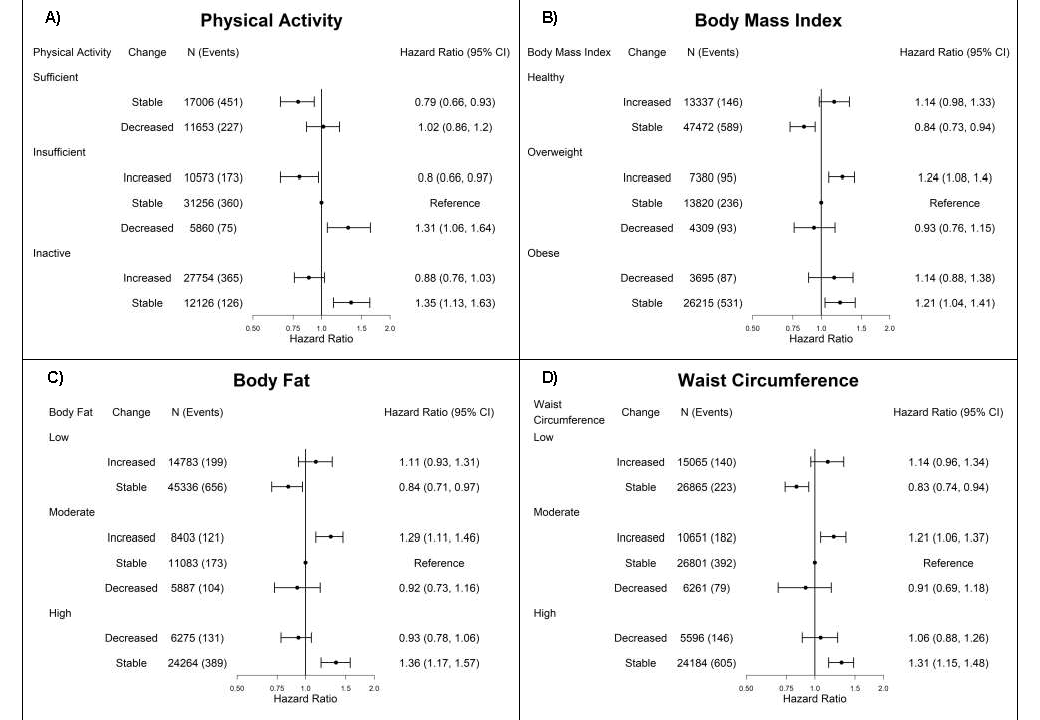
Supplemental Figure 13: Independent association for physical activity, body fat, body mass index, and waist circumference changes with cancer mortality with adjustment for hypertension and diabetes, and mutual adjustment for physical activity and adiposity.

Adjusted for: age, sex, physical activity group (for adiposity exposures), body mass index group (for physical activity as the exposure), smoking status, alcohol consumption, sleep duration, diet (fruits and vegetables consumption), education, diabetes, and hypertension.


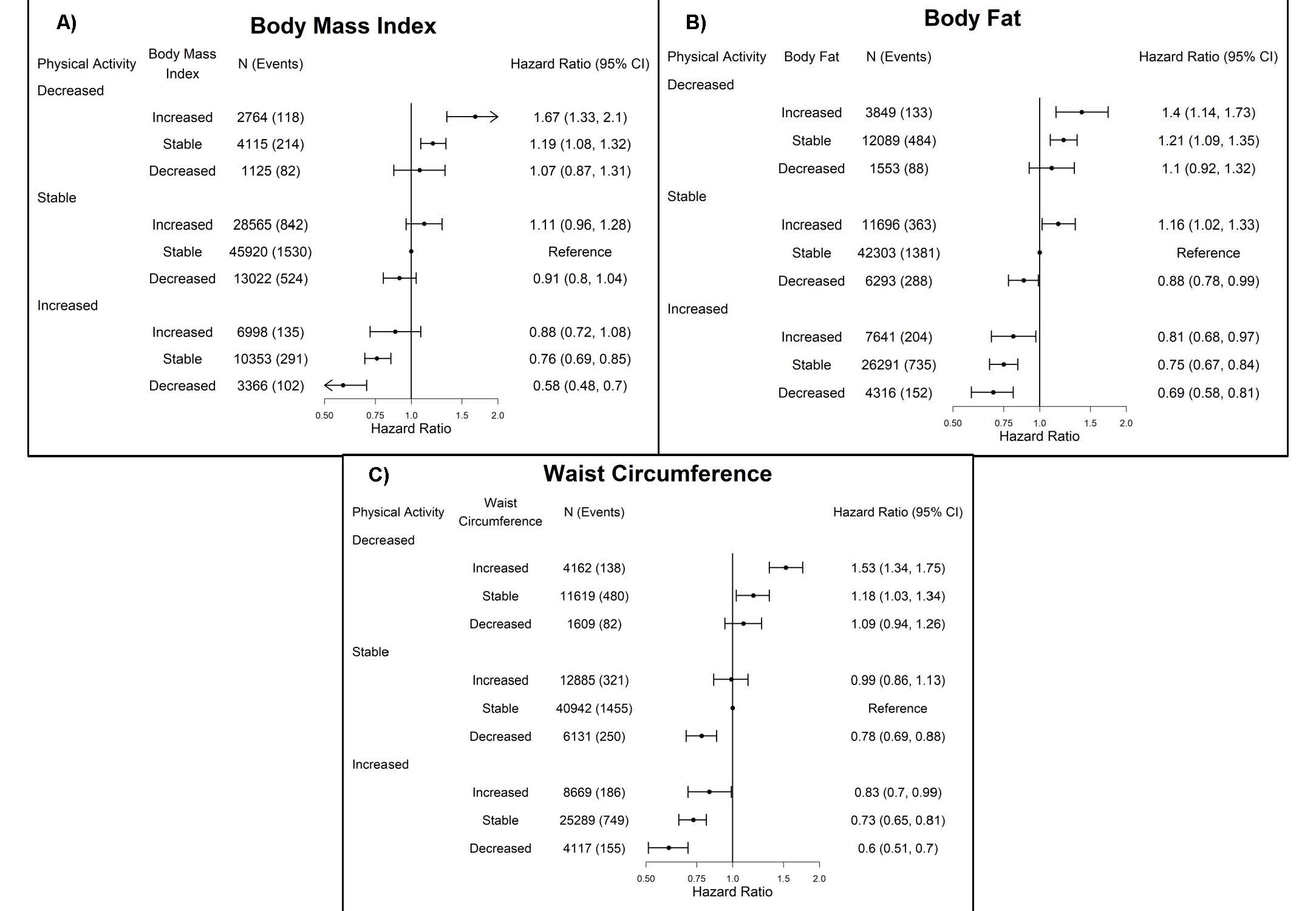
Supplemental Figure 14: Joint association of physical activity and adiposity changes with all-cause mortality with adjustment for hypertension and diabetes

Adjusted for: age, sex, baseline physical activity, baseline adiposity (body fat percentage, body mass index, or waist circumference), smoking status, alcohol consumption, sleep duration, diet (fruits and vegetables consumption), education, diabetes, and hypertension


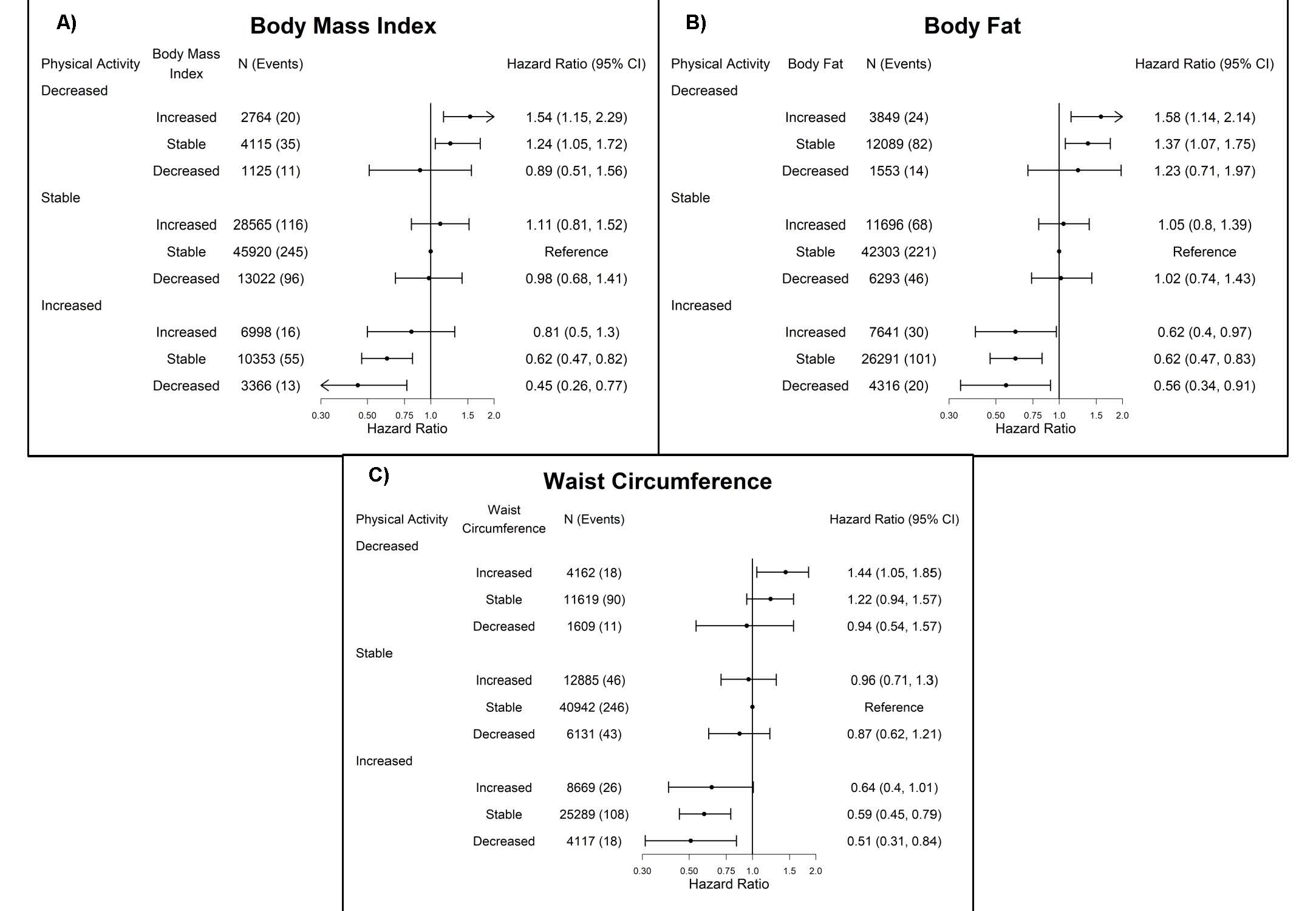
Supplemental Figure 15: Joint association of physical activity and adiposity changes with cardiovascular disease mortality with adjustment for hypertension and diabetes

Adjusted for: age, sex, baseline physical activity, baseline adiposity (body fat percentage, body mass index, or waist circumference), smoking status, alcohol consumption, sleep duration, diet (fruits and vegetables consumption), education, diabetes, and hypertension


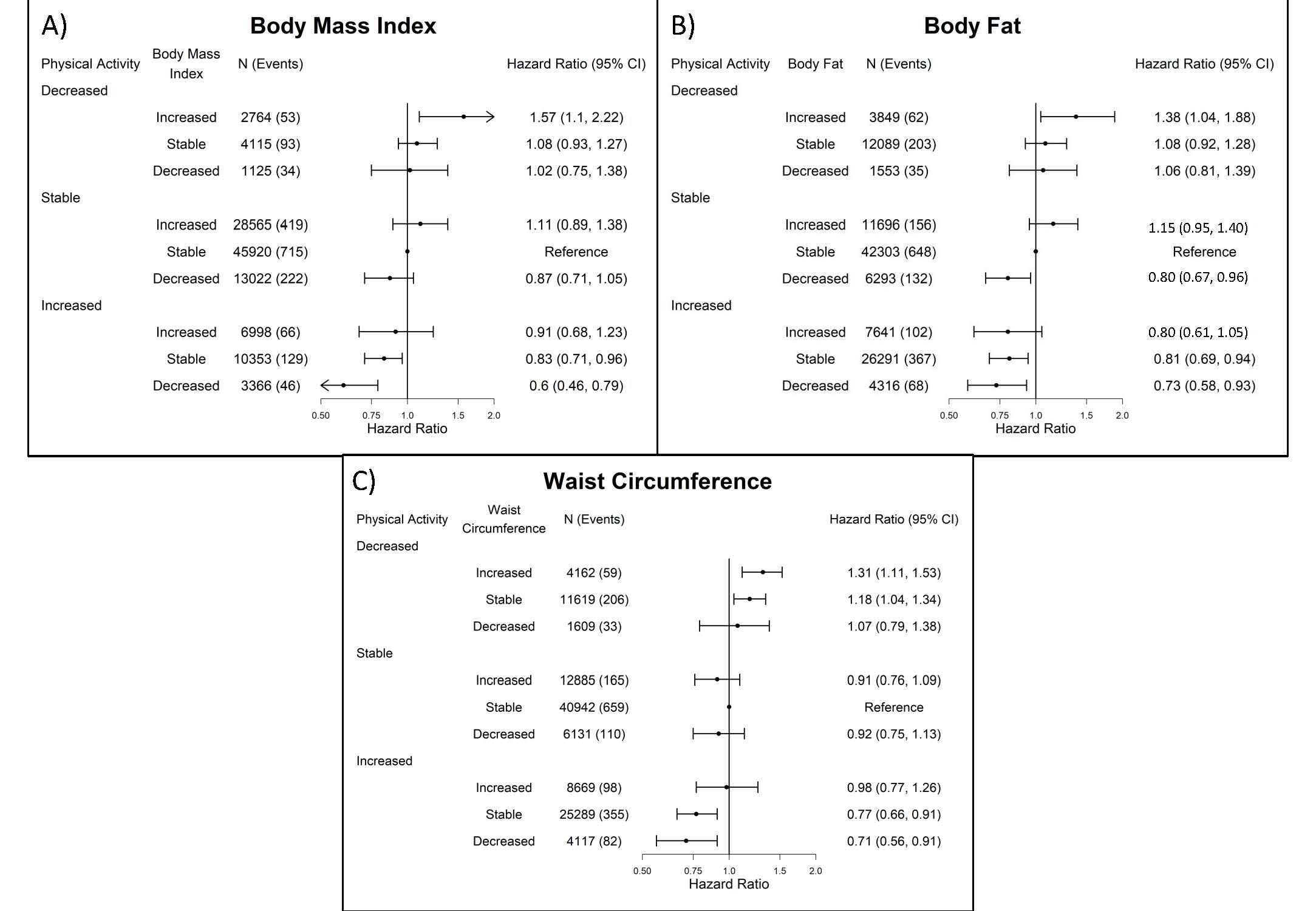
Supplemental Figure 16: Joint association of physical activity and adiposity changes with cancer mortality with adjustment for hypertension and diabetes

All results are adjusted for: age, sex, baseline physical activity, baseline adiposity (body fat percentage, body mass index, or waist circumference), smoking status, alcohol consumption, sleep duration, diet (fruits and vegetables consumption), education, diabetes, and hypertension

Supplemental Text: MJ Cohort physical activity questionnaire, construct validity, and face validity

Physical activity was measured using two versions of the MJ Cohort PA questionnaire between 1998 to 2014. Between 1998 and 2008 (version 1), the questionnaire asked participants to report the types and intensities of weekly physical activities they engaged in the previous month. Four intensity categories of exercises were provided: light (e.g., walking), moderate (e.g., brisk walking), medium-vigorous (e.g., jogging), or high-vigorous (e.g., running). Then, participants reported the duration per week spent on each of these activities in the previous month. Between 2009 and 2013 (version 2), participants reported the frequency and duration they spent on two most prominent activity types and intensities in the last two weeks. They were asked to report the frequency and duration they spent on these activities. For physical activity changes, we only included participants who had a follow up assessment using the same questionnaire version as their baseline assessment. The questions and response options for both versions are provided below. In both versions, for individuals who indicated activities in more than one intensity category, a weighted MET value was assigned, dependent on the length of time engaged in each category, as previously described by Wen et al (Lancet 2011; 378: 1244-53).

MJ physical activity questionnaire 1998-2008

| **What kind of physical activities do you usually do? (Multiple choice)** | - Light exercise: gardening, sweeping the floor, mopping the floor, golf, baseball, light aerobics, dancing (regular), biking (slow speed) - Medium exercise: basketball, volleyball, table tennis, badminton, dancing (intensive), swimming (as wished), brisk walking - Heavy exercise: jogging (8 kilometers per hour), mountain climbing, climbing the stairs, swimming (freestyle and breaststroke) - Intensive exercise: running (12 kilometers per hour), jump rope, rowing, swimming (butterfly), speed skating |
| --- | --- |
| **How much time do you devote to each activity in the last four weeks** | - None or less than 1 h/week - 1-2 h/week - 3-4 h/week - 5-6 h/week - over 7 h/week |

MJ physical activity questionnaire 2009-2014

| **What kind of physical activities do you usually do? (Select up to 2 choices)** | - Light exercise: gardening, sweeping the floor, mopping the floor, golf, baseball, light aerobics, dancing (regular), biking (slow speed) - Medium exercise: basketball, volleyball, table tennis, badminton, dancing (intensive), swimming (as wished), brisk walking - Heavy exercise: jogging (8 kilometers per hour), mountain climbing, climbing the stairs, swimming (freestyle and breaststroke) - Intensive exercise: running (12 kilometers per hour), jump rope, rowing, swimming (butterfly), speed skating |
| --- | --- |
| **How often did you do each activity during the last two weeks?** | - 2-3 times a day - once a day - once every 2-3 days - once a week - none or rarely |
| **How many hours did you spend on each activity during the last two weeks?** | - <0.5 h - 0.5-1 h - 1-2 h - over 2 h |

Construct validity: physical activity associations with cholesterol ratio (total cholesterol / HDL) in full sample of available participants

| Physical activity | N / events | β (95% CI) | P-trend |
| --- | --- | --- | --- |
| 1998-2008 |  |  |  |
| Inactive | 139,563 / 5,582 | Reference | *p<0.01* |
| Insufficient | 74,833 / 4,461 | -0.07 (-0.08, -0.06) |  |
| Sufficient | 74, 364 / 2,995 | -0.16 (-0.17, -0.15) |  |
| 2009-2013 |  |  |  |
| Inactive | 32,074 / 1,411 | Reference | *p<0.01* |
| Insufficient | 35,975 / 1,475 | -0.06 (-0.08, -0.05) |  |
| Sufficient | 21,549 / 948 | -0.16 (-0.18, -0.15) |  |

Analysis is adjusted for age and sex. Results are from generalised linear modelling with gaussian distribution, identity link, and robust standard errors. Sample includes baseline physical activity groups from all eligible participants with complete physical activity, age, sex, and cholesterol ratio data.


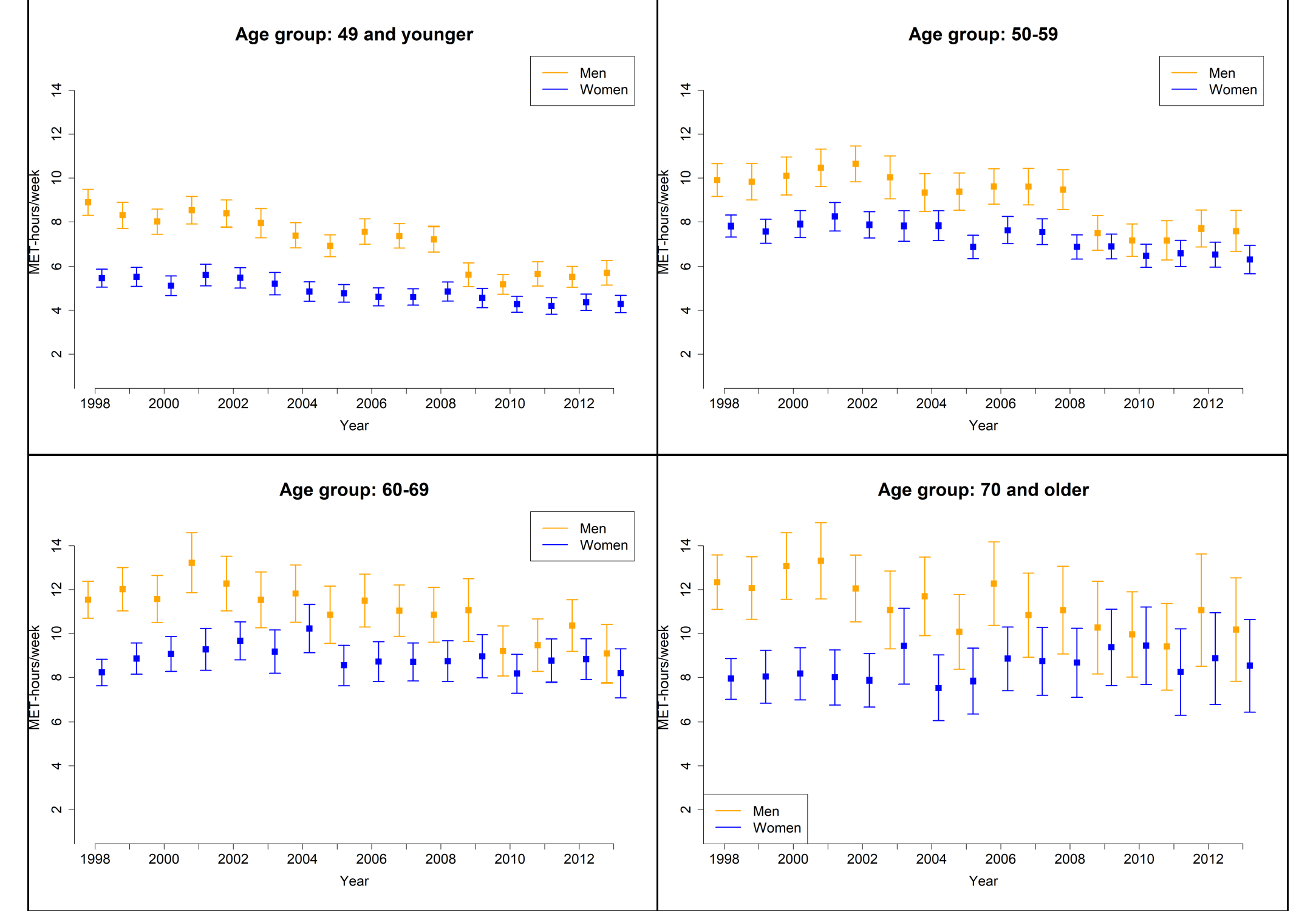
Face validity: physical activity MET-hrs/week by age and sex from 1998 to 2013

Sample size for: 49 and younger= male:35,811, female:34,620; 50-59 years= male: 21,812, female: 30,153; 60-69 years= male: 13,746, female: 15,342; 70 and older= male: 5,410, female: 4,893. Sample includes baseline values from all participants with complete physical activity data.

STROBE Statement—checklist of items that should be included in reports of observational studies

|  | Item No | Recommendation | Page  No |
| --- | --- | --- | --- |
| **Title and abstract** | 1 | (*a*) Indicate the study’s design with a commonly used term in the title or the abstract | 1,2 |
|  |  | (*b*) Provide in the abstract an informative and balanced summary of what was done and what was found | 1,2 |
| Introduction | | | |
| Background/rationale | 2 | Explain the scientific background and rationale for the investigation being reported | 3,4 |
| Objectives | 3 | State specific objectives, including any prespecified hypotheses | 4 |
| Methods | | | |
| Study design | 4 | Present key elements of study design early in the paper | 4-7 |
| Setting | 5 | Describe the setting, locations, and relevant dates, including periods of recruitment, exposure, follow-up, and data collection | 4 |
| Participants | 6 | (*a*) *Cohort study*—Give the eligibility criteria, and the sources and methods of selection of participants. Describe methods of follow-up  *Case-control study*—Give the eligibility criteria, and the sources and methods of case ascertainment and control selection. Give the rationale for the choice of cases and controls  *Cross-sectional study*—Give the eligibility criteria, and the sources and methods of selection of participants | 4, supplemental figure 1 |
|  |  | (*b*) *Cohort study*—For matched studies, give matching criteria and number of exposed and unexposed  *Case-control study*—For matched studies, give matching criteria and the number of controls per case | NA |
| Variables | 7 | Clearly define all outcomes, exposures, predictors, potential confounders, and effect modifiers. Give diagnostic criteria, if applicable | 4-7 |
| Data sources/ measurement | 8* | For each variable of interest, give sources of data and details of methods of assessment (measurement). Describe comparability of assessment methods if there is more than one group | 4-7, supplemental text |
| Bias | 9 | Describe any efforts to address potential sources of bias | 4,7,8 |
| Study size | 10 | Explain how the study size was arrived at | Supplemental Figure 1 |
| Quantitative variables | 11 | Explain how quantitative variables were handled in the analyses. If applicable, describe which groupings were chosen and why | 4,5,7 |
| Statistical methods | 12 | (*a*) Describe all statistical methods, including those used to control for confounding | 7,8 |
|  |  | (*b*) Describe any methods used to examine subgroups and interactions | 7,8 |
|  |  | (*c*) Explain how missing data were addressed | NA |
|  |  | (*d*) *Cohort study*—If applicable, explain how loss to follow-up was addressed  *Case-control study*—If applicable, explain how matching of cases and controls was addressed  *Cross-sectional study*—If applicable, describe analytical methods taking account of sampling strategy | NA |
|  |  | (*e*) Describe any sensitivity analyses | NA |

| Results | | | |
| --- | --- | --- | --- |
| Participants | 13* | (a) Report numbers of individuals at each stage of study—eg numbers potentially eligible, examined for eligibility, confirmed eligible, included in the study, completing follow-up, and analysed | Supplemental Figure 1 |
|  |  | (b) Give reasons for non-participation at each stage | Supplemental Figure 1 |
|  |  | (c) Consider use of a flow diagram | Supplemental Figure 1 |
| Descriptive data | 14* | (a) Give characteristics of study participants (eg demographic, clinical, social) and information on exposures and potential confounders | Table 1, supplemental table 3 |
|  |  | (b) Indicate number of participants with missing data for each variable of interest | Supplemental Figure 1 |
|  |  | (c) *Cohort study*—Summarise follow-up time (eg, average and total amount) | 8 |
| Outcome data | 15* | *Cohort study*—Report numbers of outcome events or summary measures over time | Figures 1-4, supplemental figures 2-16 |
|  |  | *Case-control study—*Report numbers in each exposure category, or summary measures of exposure | *NA* |
|  |  | *Cross-sectional study—*Report numbers of outcome events or summary measures | *NA* |
| Main results | 16 | (*a*) Give unadjusted estimates and, if applicable, confounder-adjusted estimates and their precision (eg, 95% confidence interval). Make clear which confounders were adjusted for and why they were included | Table 1, Figures 1-4 |
|  |  | (*b*) Report category boundaries when continuous variables were categorized | NA |
|  |  | (*c*) If relevant, consider translating estimates of relative risk into absolute risk for a meaningful time period | NA |
| Other analyses | 17 | Report other analyses done—eg analyses of subgroups and interactions, and sensitivity analyses | 12-13, Supplemental Figures 2-16 |
| Discussion | | | |
| Key results | 18 | Summarise key results with reference to study objectives | 13-16 |
| Limitations | 19 | Discuss limitations of the study, taking into account sources of potential bias or imprecision. Discuss both direction and magnitude of any potential bias | 16-17 |
| Interpretation | 20 | Give a cautious overall interpretation of results considering objectives, limitations, multiplicity of analyses, results from similar studies, and other relevant evidence | 13-18 |
| Generalisability | 21 | Discuss the generalisability (external validity) of the study results | NA |
| Other information | | | |
| Funding | 22 | Give the source of funding and the role of the funders for the present study and, if applicable, for the original study on which the present article is based | 1 |
